# Hemokinin-1 induces transcriptomic alterations in pain-related signaling processes in rat primary sensory neurons independent of NK1 tachykinin receptor activation

**DOI:** 10.3389/fnmol.2023.1186279

**Published:** 2023-10-27

**Authors:** Krisztina Takács-Lovász, Timea Aczél, Éva Borbély, Éva Szőke, Lilla Czuni, Péter Urbán, Attila Gyenesei, Zsuzsanna Helyes, József Kun, Kata Bölcskei

**Affiliations:** ^1^Department of Pharmacology and Pharmacotherapy, Medical School and Centre for Neuroscience, University of Pécs, Pécs, Hungary; ^2^National Laboratory for Drug Research and Development, Budapest, Hungary; ^3^Hungarian Research Network, PTE HUN-REN Chronic Research Group, Budapest, Hungary; ^4^Szentágothai Research Centre, Bioinformatics Research Group, Genomics and Bioinformatics Core Facility, University of Pécs, Pécs, Hungary; ^5^PharmInVivo Ltd., Pécs, Hungary

**Keywords:** trigeminal ganglion, cell culture, transcriptomics, pain, migraine, Mrgpr-ANTXR2, tac4

## Abstract

The tachykinin hemokinin-1 (HK-1) is involved in immunological processes, inflammation, and pain. Although the neurokinin 1 receptor (NK1R) is described as its main target, several effects are mediated by currently unidentified receptor(s). The role of HK-1 in pain is controversial, depending on the involvement of peripheral and central sensitization mechanisms in different models. We earlier showed the ability of HK-1 to activate the trigeminovascular system, but the mechanisms need to be clarified. Therefore, in this study, we investigated HK-1-induced transcriptomic alterations in cultured rat trigeminal ganglion (TRG) primary sensory neurons. HK-1 was applied for 6 or 24 h in 1 μM causing calcium-influx in these neurons, 500 nM not inducing calcium-entry was used for comparison. Next-generation sequencing was performed on the isolated RNA, and transcriptomic changes were analyzed to identify differentially expressed (DE) genes. Functional analysis was performed for gene annotation using the Gene Ontology (GO), Kyoto Encyclopedia of Genes and Genomes (KEGG), and Reactome databases. NK1R and Neurokinin receptor 2 (NK2R) were not detected. Neurokinin receptor 3 (NK3R) was around the detection limit, which suggests the involvement of other NKR isoforms or other receptors in HK-1-induced sensory neuronal activation. We found protease-activated receptor 1 (PAR1) and epidermal growth factor receptor (EGFR) as DE genes in calcium signaling. The transmembrane protein anthrax toxin receptor 2 (ANTXR2), a potential novel pain-related target, was upregulated. Acid-sensing ion channel 1; 3 (Asic1,3), N-methyl-D-aspartate (NMDA) and alpha-amino-3-hydroxy-5-methyl-4-isoxazolepropionic acid (AMPA) glutamate receptors decreased, myelin production and maintenance related genes (Mbp, Pmp2, Myef2, Mpz) and GNDF changed by HK-1 treatment. Our data showed time and dose-dependent effects of HK-1 in TRG cell culture. Result showed calcium signaling as altered event, however, we did not detect any of NK receptors. Presumably, the activation of TRG neurons is independent of NK receptors. ANTXR2 is a potential new target, PAR-1 has also important role in pain, however their connection to HK-1 is unknown. These findings might highlight new targets or key mediators to solve how HK-1 acts on TRG.

## 1. Introduction

Tachykinins represent a neuropeptide family widely distributed in the body. The first members of this family were substance P (SP), neurokinin A (NKA), derived from the preprotachykinin A/Tac1 gene, and neurokinin B (NKB), encoded by the preprotachykinin B/Tac3 gene (Borbély and Helyes, 2017). Other members of this family have also been identified: neuropeptide K (NPK) (Roth et al., 1985), neuropeptide γ (NPγ) (Kage et al., 1988), hemokinin-1 (HK-1), and endokinins (EKs) (Kurtz et al., 2002). HK-1 encoded by the preprotachykinin C/Tac4 gene was discovered 20 years ago in B lymphocytes (Zhang et al., 2000), and similar to SP, it was shown in several immune cell types, including myeloid (polymorphonuclear granulocytes and eosinophils), lymphoid, dendritic cells, neurons, and microglia (Borbély and Helyes, 2017). This widespread expression of HK-1 in diverse cell types might be related to a broad range of physiological and pathophysiological functions by activating a multitude of signaling pathways, which are currently not understood. HK-1 induces a variety of physiological and pathophysiological functions in the immune and hematopoietic systems, gastrointestinal tract, airways, cardiovascular, endocrine, and neural systems, bone, joints, and cancer. The role of HK-1 in pain is contradictory and likely to be concentration/dose dependent (Borbély and Helyes, 2017). In mice, centrally administered HK-1 caused scratching (Borbély and Helyes, 2017), licking, and biting at low doses (Watanabe et al., 2010). Intrathecally injected HK-1 was analgesic in nanomolar concentrations in an NK1- or opioid receptor-dependent manner, but hyperalgesic in picomolar concentrations (Fu et al., 2005).

Tachykinins possess a differential affinity for the three tachykinin receptors. HK and EKs have the highest affinity for NK1R, similar to SP (Satake et al., 2013; Steinhoff et al., 2014). They are all G protein-coupled receptors of the rhodopsin family. Signaling through the NK receptors is complex with multiple directions: (1) G_q_-related: activation of phospholipase C, resulting in inositol trisphosphate and diacylglycerol formation, mobilization of Ca^2+^ from intracellular stores, and activation of protein kinase C; (2) G_s_-related activation of adenylyl cyclase, resulting in the cAMP formation and protein kinase A (PKA) activation; or (3) activation of phospholipase A2 and arachidonic acid production (Steinhoff et al., 2014; Garcia-Recio and Gascón, 2015). The existence of a specific receptor for HK-1 is currently intensively investigated (Duffy et al., 2003). It was shown that an NK1R antagonist did not inhibit pain caused by HK-1 in different concentrations, suggesting a specific HK-1 target/receptor (Watanabe et al., 2010). NK1R antagonists were developed as analgesic drug candidates, but they failed in human pain conditions. This might be due to different NK1R splice variants linked to distinct binding and activation mechanisms and/or a currently unidentified receptor (Zhang et al., 2000; Borbély and Helyes, 2017; Hunyady et al., 2019; Borbély et al., 2020). Our research group earlier showed that HK-1 mediated chronic adjuvant-induced inflammation and related pain (Borbély et al., 2013) and neuropathic and neurogenic inflammatory hyperalgesia via NK1 receptor activation (Hunyady et al., 2019). Interestingly, we recently demonstrated that unlike SP, HK-1 activates primary sensory neurons by inducing calcium influx via an NK1R-independent mechanism (Borbély et al., 2020). NK1R was shown to interact with the Mas-related G protein receptors (Mrgr) in certain pain-related mechanisms (Zhou et al., 2015). The human Mrgprx2, which is an ortholog of the rat Mrgprb5, was described as a potential target for HK-1 in mast cells to cause degranulation (Manorak et al., 2018). However, naturally occurring variants of Mrgprx2 lose HK-1 binding ability, showing the high importance of receptor variants (Alkanfari et al., 2018).

Based on a broad range of data demonstrating proinflammatory and pronociceptive functions of HK-1 in different organs partially independently of NK1 receptor activation (Hunyady et al., 2019; Borbély et al., 2020), our main aim was to identify the molecular mechanisms responsible for these actions. Our recent results demonstrated NK1R-independent calcium influx in cultured trigeminal ganglion cells induced by 1 μM, but not 500 nM HK-1 (Borbély et al., 2020). In the present study, we investigated the concentration- and treatment duration-dependent intracellular signaling mechanisms and pathways in these primary sensory neurons using an unbiased transcriptomic approach.

## 2. Materials and methods

### 2.1. Primary cultures of TG neurons and treatment protocols

Primary cell cultures of TG neuron cells were extracted from 1–4-day-old Wistar rat pups as described earlier (Szoke et al., 2000). TG cells were dissected and washed in several steps. Afterward, TG cells were plated on poly-D-lysin-coated glass coverslips and grown in a nutrient-supplemented medium for the experiment. The earlier washing steps and the cell culture medium recipe are detailed elsewhere (Takács-Lovász et al., 2022). According to an earlier calcium influx, cell cultures were treated with HK-1 (Sigma Aldrich, solved in culture media) in two concentrations: 500 nM (no evoked calcium influx) and 1 μM [evoked calcium influx in an earlier study (Takács-Lovász et al., 2022)]. Untreated cultures were used as controls. After 6 h and 24 h of HK-1 administration, samples were collected for RNA isolation. Except for the HK-1 500 nM 24 h condition, which was repeated in duplicates, other conditions were repeated in triplicate.

### 2.2. Illumina library preparation and sequencing

In order to provide a complete gene expression profile, RNA sequencing was performed (Fang and Cui, 2011). The library for Illumina sequencing was made using the QuantSeq 30 mRNA-Seq Library Prep Kit FWD for Illumina (Lexogen, Vienna, Austria). A total of 400 ng of total RNA was used for first-strand cDNA generation using an oligodT primer followed by RNA removal. The second strand synthesis was followed by random priming, and the products were purified with magnetic beads. Finally, the libraries were amplified and barcoded using PCR. All libraries were quality-checked on the TapeStation 4200 (Agilent Technologies, Santa Clara, CA, USA) to examine if adapter dimers formed during PCR. The QuantSeq libraries were sequenced using the Illumina NextSeq550 platform to produce 75 bp single-end reads.

### 2.3. Bioinformatics

The sequencing reads were aligned against the *Rattus norvegicus* reference genome (Rnor 6.0 Ensembl release) with STAR v2.5.3a (Dobin et al., 2013). Following alignment, reads were associated with known protein-coding genes, and the number of reads aligned within each gene was counted using HTSeq library v0.11.1 (Anders et al., 2015). Gene count data were normalized using the trimmed mean of M values (TMM) normalization method of the edgeR R/Bioconductor package (v3.28, R v3.6.0, Bioconductor v3.9) (Robinson et al., 2010). Data were further log-transformed using the voom approach for statistical evaluation (Law et al., 2014) in the limma package (Ritchie et al., 2015). Fold change (FC) values between the compared groups resulting from the linear modeling process and moderated *t*-test *p-*values relative to a minimum required fold change threshold were calculated by the limma package. When determining differentially expressed (DE) genes, filtering thresholds were set to at least FC 1,2 and *p*-value 0.05 when the HK-1 1 μM 24 h and HK-1 500 nM 6 h treatments were compared to the untreated control group, and to FC 1.3 and *p*-value 0.05 for the HK-1 1 μM 6 h vs. untreated control, and to FC 2 and *p*-value 0.05 for HK-1 500 nM 24 h vs. untreated control group comparison. Normalized counts were represented as transcripts per million (TPM) values. Functional analysis (annotations of genes) was prepared using the Gene Ontology (GO), Kyoto Encyclopedia of Genes and Genomes (KEGG), and Reactome databases. Detection of functional enrichment was performed in the differentially expressed gene list (DE list enrichment: Fisher's exact test for GO, hypergeometric test for KEGG and Reactome) and toward the top of the list when all genes have been ranked according to the evidence for being differentially expressed (ranked list enrichment: non-parametric Kolmogorov–Smirnov test for GO and KEGG, hypergeometric test for Reactome) applying the topGO v2.37.0, ReactomePA v1.30.0, and gage v2.36.0 packages. The pathview package v1.26.0 (Luo and Brouwer, 2013) was used to visualize mapping data to KEGG pathways. GO terms were merged using the Revigo tool (tiny resulting list set up) for extracting the most relevant terms.

### 2.4. Validation of gene expression fold changes by RT-qPCR

To validate certain up and downregulated genes together with unaltered ones determined by RNA sequencing, 200 ng RNA per sample was used for reverse transcription with the High-Capacity cDNA Reverse Transcription Kit (Thermo Fisher Scientific). Primers were designed with Primer-BLAST (https://www.ncbi.nlm.nih.gov/tools/primer-blast/) for the genes selected on the basis of the transcriptomics results [nuclear receptor subfamily 4 group A member 1 (Nr4a1), solute carrier family 25 member 5 (Slc25a5), fibroblast growth factor receptor 1 (Fgfr1), heat shock protein HSP 90-alpha (Hsp90aa1), integrin subunit alpha 4 (Itga4), NADH: ubiquinone oxidoreductase subunit B6 (Ndufb6), F2r/PAR1, G protein subunit beta 2 (Gnb2), G protein subunit alpha I1 (Gnai1), fibroblast growth factor 5 (Fgf5)], and they are demonstrated in Supplementary Table S1. The reactions were carried out with SensiFAST SYBR^®^ Lo-ROX mix (Meridian BioScience) reagents in triplicate using 1 μl of cDNA per well in a Bio-Rad CFX96 real-time cycler. The amplification program was as follows: 95 °C for 120 s, 40 cycles at 95 °C for 5 s, 58 °C for 10s, and 60 °C for 30 s. The geometric mean of the CT values for the two housekeeping genes was determined to obtain the ΔCT for each gene of interest. The ΔΔCT values were calculated by subtracting the ΔCT value of the control sample from the ΔCT of the treated sample. Relative fold changes in gene expression were calculated using the comparative 2–ΔΔCt method (Livak and Schmittgen, 2001).

## 3. Results

### 3.1. DE genes

Extracting the most important and relevant DE genes helps to understand the effects and intracellular signaling mechanisms of HK-1. In average rank, the p and FC values were taken into account shows the most relevant ranked gene list for all groups (Table 1). After 24 h of response to 1 μM HK-1, which we previously showed induces calcium influx in trigeminal primary sensory neurons, acid-sensing ion channel subunit 3 (Asic3), Glutamate ionotropic receptor NMDA type subunit 1 (Grin1), and C-C motif chemokine ligand 7 (Ccl7) were downregulated. At the earlier, 6 h timepoint, 1 μM HK-1 downregulated Slc25a5, while upregulating myelin-associated glycoprotein (Mag). For 6 h results, Mag, Itga4, and the LBH regulator of the Wnt signaling pathway (Lbb) were at the top of the list and upregulated independently of concentration.

**Table 1:** Collection of the first 30 average ranked genes with FC value for all cases.

**avgRank**	**HK-1 1**μ**M_24h**			**HK-1 1**μ**M_6h**			**HK-1 500 nM 24h**			**HK-1 500 nM 6h**		
	**Description**	**Gene**	**FC**	**Description**	**Gene**	**FC**	**Description**	**Gene**	**FC**	**Description**	**Gene**	**FC**
1	Receptor (chemosensory) transporter protein 4	Rtp4	−5.0	Integrin subunit alpha 4	Itga4	7.5	Nuclear receptor subfamily 4, group A, member 1	Nr4a1	30.2	H1.1 linker histone, cluster member	H1f1	11.9
2	Limb and CNS expressed 1	Lix1	−4.1	Mitogen-activated protein kinase kinase kinase 20	Map3k20	4.4	Solute carrier family 25 member 5	Slc25a5	15.3	Integrin subunit alpha 4	Itga4	4.4
3	Potassium voltage-gated channel-interacting protein 1	Kcnip1	−3.9	LBH regulator of WNT signaling pathway	Lbh	4.1	Cellular communication network factor 1	Ccn1	19.5	Betacellulin	Btc	3.6
4	Glycine receptor, beta	Glrb	−3.2	H1.1 linker histone, cluster member	H1f1	15.1	Ring finger protein 5	Rnf5	28.8	Insulin-like growth factor 2 mRNA binding protein 3	Igf2bp3	3.9
5	Transmembrane protein 45b	Tmem45b	−3.3	Solute carrier family 25 member 5	Slc25a5	−4.5	U2 small nuclear RNA auxiliary factor 2	U2af2	−9.3	LBH regulator of WNT signaling pathway	Lbh	2.7
6	Ras-like without CAAX 2	Rit2	−2.8	H1.4 linker histone, cluster member	H1f4	3.8	Basic transcription factor 3	Btf3	10.1	Cysteine and glycine-rich protein 2	Csrp2	3.3
7	Chondromodulin	Cnmd	−5.1	tropomyosin 4	Tpm4	3.7	Ribosomal protein S13	Rps13	11.6	Exocyst complex component 1 like	Exoc1l	−3.2
8	Radical S-adenosyl methionine domain containing 2	Rsad2	−4.6	shootin 1	Shtn1	3.9	Dihydropyrimidinase-like 3	Dpysl3	−6.6	H1.4 linker histone, cluster member	H1f4	2.6
9	Diacylglycerol kinase, gamma	Dgkg	−2.9	La ribonucleoprotein 4B	Larp4b	4.0		AC141489.1	6.9	H1.5 linker histone, cluster member	H1f5	6.5
10	RT1 class II, locus Db1	RT1-Db1	−2.4	High mobility group AT-hook 1	Hmga1	4.2	La ribonucleoprotein 4B	Larp4b	−6.0	Serine protease 12	Prss12	2.6
11	Expressed sequence AI593442	LOC100125362	−2.7	Dihydropyrimidinase-like 3	Dpysl3	3.6	Cilia and flagella associated protein 20	Cfap20	7.2	Mitochondrially encoded NADH dehydrogenase 6	Mt-nd6	−2.4
12	Inhibin subunit beta B	Inhbb	−2.7	BMP2 inducible kinase	Bmp2k	3.7	Hexokinase 1	Hk1	−6.9	Catenin beta 1	Ctnnb1	5.6
13	Elastin	Eln	−2.5	TIAM Rac1 associated GEF 1	Tiam1	3.9	Guanine nucleotide-binding protein G(I)/G(S)/G(O) subunit gamma-5	LOC108349548	5.4	Ly6/Plaur domain containing 1	Lypd1	3.0
14	Acid-sensing ion channel subunit 3	Asic3	−2.9	U2 small nuclear RNA auxiliary factor 2	U2af2	5.0	Mesoderm specific transcript	Mest	−5.8	Apolipoprotein E	Apoe	−2.5
15	Guanosine monophosphate reductase	Gmpr	−2.3	Serine/threonine kinase 10	Stk10	3.6	Sorting nexin 7	Snx7	−5.8	shootin 1	Shtn1	2.6
16	Dispatched RND transporter family member 2	Disp2	−2.2	H1.2 linker histone, cluster member	H1f2	6.4	ANKH inorganic pyrophosphate transport regulator	Ankh	−6.9		AABR07035787.1	2.5
17	Glutamate ionotropic receptor NMDA type subunit 1	Grin1	−2.3	Heat shock protein family A (Hsp70) member 9	Hspa9	3.3	Translin-associated factor X	Tsnax	7.9	Scm-like with four mbt domains 2	Sfmbt2	4.1
18	Homer scaffold protein 3	Homer3	−2.2	Myotubularin-related protein 2	Mtmr2	4.1	Pyruvate kinase M1/2	Pkm	−8.5	Cysteine and glycine-rich protein 1	Csrp1	2.4
19	Transmembrane protein 72	Tmem72	−2.8	ANKH inorganic pyrophosphate transport regulator	Ankh	4.3	Signal recognition particle 9	Srp9	4.8	Lipoprotein lipase	Lpl	−2.3
20	Microsomal glutathione S-transferase 3	Mgst3	−4.1	RAP2A, member of the RAS oncogene family	Rap2a	3.2	Formin binding protein 1	Fnbp1	−6.4	DEAD (Asp-Glu-Ala-Asp) box polypeptide 3	Ddx3	3.8
21	Protein phosphatase 2C-like domain containing 1	Pp2d1	−2.2	Myb/SANT DNA binding domain containing 2	Msantd2	4.5	Discoidin, CUB, and LCCL domain containing 2	Dcbld2	−4.8	Lysophosphatidic acid receptor 4	Lpar4	3.6
22	Gap junction protein, beta 1	Gjb1	−2.2	Filamin A interacting protein 1-like	Filip1l	3.7		AABR07028615.1	−5.2	Insulin-like growth factor 2	Igf2	2.4
23	C-C motif chemokine ligand 7	Ccl7	−2.4	G protein subunit beta 2	Gnb2	−6.5	RNA polymerase II subunit D	Polr2d	10.5	CUE domain containing 2	Cuedc2	2.5
24	Pleckstrin homology and RhoGEF domain containing G6	Plekhg6	−2.4	Nebulin	Neb	3.3		AABR07057617.1	6.3	Adrenomedullin	Adm	−2.4
25	Synaptotagmin 6	Syt6	−2.6	Jumonji and AT-rich interaction domain containing 2	Jarid2	3.6	Golgin A7	Golga7	6.8	Myelin-associated glycoprotein	Mag	2.8
26	Frizzled class Receptor 1	Fzd1	2.0	Cellular communication network factor 1	Ccn1	−3.6	Selenoprotein F	Selenof	5.1	Calponin 1	Cnn1	3.3
27	rabphilin 3A	Rph3a	−2.4	Transcription activation suppressor family member 2	Tasor2	3.1	cystatin B	Cstb	5.4	Capping actin protein, gelsolin like	Capg	2.8
28	Methionine sulfoxide reductase B2	Msrb2	−2.4	Sprouty-related, EVH1 domain containing 1	Spred1	3.7	host cell factor C1	Hcfc1	−7.4	Oxysterol binding protein 2	Osbp2	2.3
29	Solute carrier family 16, member 13	Slc16a13	2.0	Myelin-associated glycoprotein	Mag	4.0	Protocadherin gamma subfamily C, 3	Pcdhgc3	−5.4	Cyclin-dependent kinase inhibitor 1C	Cdkn1c	2.4
30	Reprimo, TP53 dependent G2 arrest mediator homolog	Rprm	−2.0	Nuclear receptor subfamily 4, group A, member 1	Nr4a1	−6.1	NADH:ubiquinone oxidoreductase subunit B6	Ndufb6	18.3	Ankyrin repeat domain 1	Ankrd1	−5.1

The 500 nM HK-1 concentration, which did not evoke calcium influx in our earlier studies, downregulated Slc25a5 at 24 h and upregulated Ndufb6. Additionally, mitochondrially encoded NADH dehydrogenase 6 (Mt-nd6) was downregulated in the HK-1 500 nM 6 h group. Supplementary Images I1–I4 show heatmaps of all DE genes for different concentrations at different sampling times.

**Figure 1:**
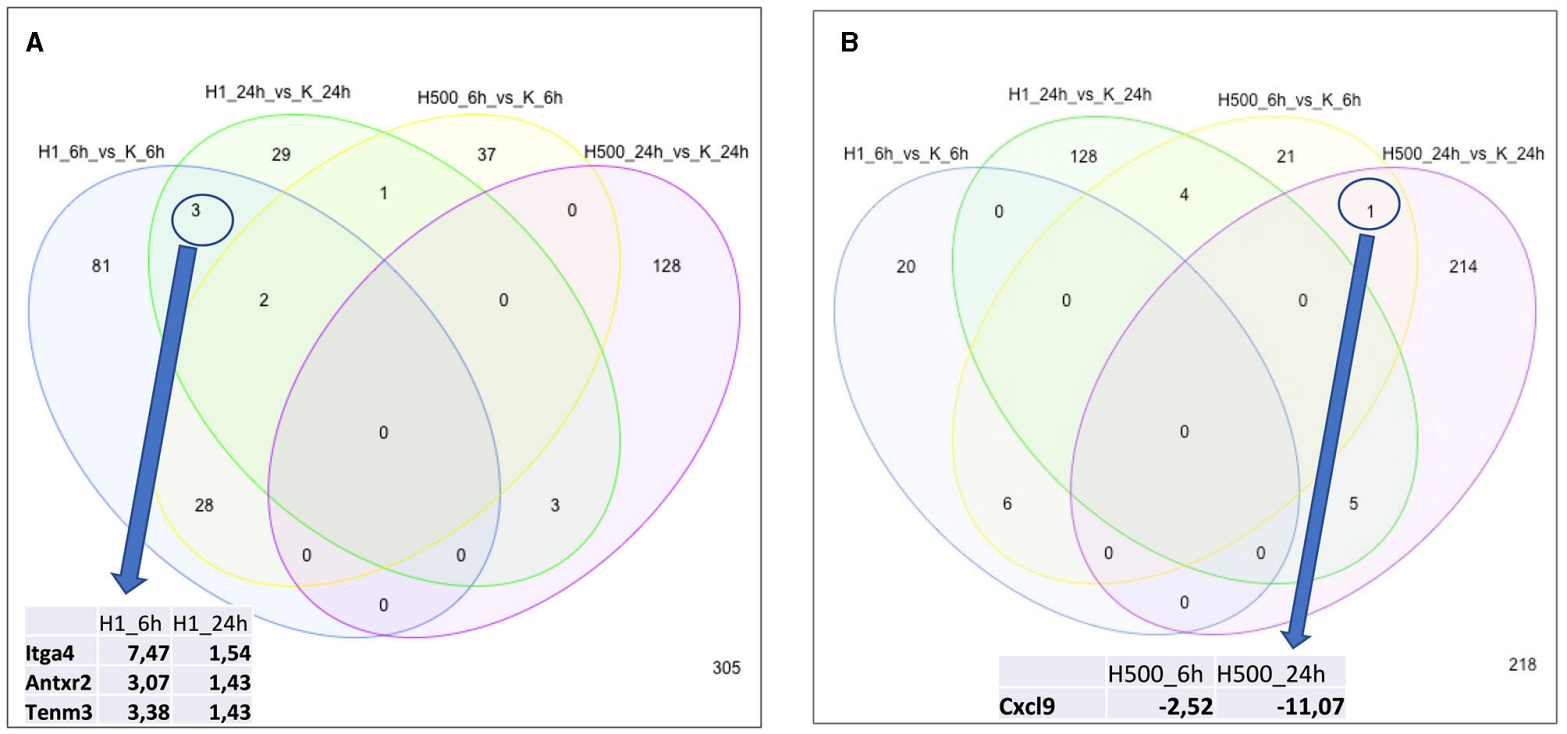
Number of DE genes for all groups. Insert tables show the FC values of the DE genes for the respective comparisons. **(A)** Panel demonstrates the upregulated genes and **(B)** shows the numbers of downregulated genes.

Similarly, altered DE genes over time (both at the 6 h and 24 h timepoints) in response to the same HK-1 concentration may yield insight into the key mechanisms and effects. Figure 1 shows the numerical representation of DE genes across all groups and a summary of different group comparisons. Most DE genes were detected in response to 500 nM HK-1 treatment after 24h, where Nr4a1, Slc25a5, F2r, Ndufb6, and Gnb2 were upregulated, which were confirmed by qPCR (FC: 1.726-; 1.783; 2.205; 2.105; 1.445, respectively). Itga4, Fgf5, and Gnai1 were upregulated, and Ndufb6, Gnb2, and F2r were downregulated 6h after 1 μM HK-1 treatment, as determined both by sequencing and qPCR (FC: 2.942; 3.652; 1.14;−1.15;−1.01;−1.08). Unaltered genes were Fgfr1 and Gnai1 according to RNA sequencing and qPCR (FC: 1.385; 1.365) 24h after 1 μM HK-1 treatment. Although qPCR confirmed all these alterations obtained with sequencing, 24 h after 500 nM HK-1 treatment, Fgfr1 changes were different with the two techniques: it was downregulated according to RNA sequencing but upregulated by PCR (FC: 1.18). The qPCR results are shown in Supplementary Table S2.

In response to 1 μM HK-1, the expression of three genes, Itga4, ANTXR cell adhesion molecule 2 (Antxr2), and teneurin transmembrane protein 3 (Tenm3), changed similarly (Figure 1). There was only one DE gene after 500 nM HK-1, Cxcl9 (C-X-C motif chemokine ligand 9), which was downregulated. Results for 24 h include less common DE genes, showing a greater concentration-dependent effect of HK-1. The common DE genes expressed in differently treated groups at the same timepoints showed 30 upregulated and 6 downregulated genes at 6 h and 3 upregulated and 5 downregulated genes at 24 h.

Common DE genes independent of the HK-1 concentration (and the calcium influx-inducing potential) at both timepoints are shown in Tables 2, 3. Antxr2 and Itga4 at 6 h were concentration-independently upregulated. Serine protease 12 (Prss12), T cell differentiation protein (Mal), and Mag were also upregulated, but the expression level of voltage-gated sodium channel beta subunit 4 (Scn4b) was decreased at 6 h after both concentrations. Regarding concentration-independent results at 24 h, frizzled class receptor 1 (Fzd1) and 3-hydroxyacyl-CoA dehydratase 2 (Hacd2) were upregulated, and rabphilin 3A (Rph3a), gamma-aminobutyric acid type A receptor alpha2 subunit (Gabra2), ryanodine receptor 2 (Ryr2) Mag, and voltage-gated sodium channel alpha subunit 1 (Scn1a) were downregulated. There were significant DE genes with potential importance in neuronal and inflammatory functions, but they are not in the top 30 ranked gene lists (Table 4). In response to 1 μM HK-1 potassium voltage-gated channel-interacting protein 4 (Kcnip4), myelin basic protein (Mbp) was downregulated and Gpr108 was upregulated at 24 h, while fibroblast growth factor 9 (Fgf9), Ndufb6, Myelin expression factor 2 (Myef2), myelin protein zero (Mpzc), and GDNF were upregulated at 6 h. Interestingly, 500 nM HK-1 downregulated PACAP, while upregulating peripheral myelin protein 2 (Pmp2), glial cell-derived neurotrophic factor (GDNF) at 6 h, transmembrane protein 128 (Tmem128), and integrin subunit alpha V (Itgav) at 24 h.

**Table 2:** Common DE genes for the 1μM and 500 nM HK-1 concentrations at 6 h. Red means upregulated, while green shows downregulated DE genes.

**geneSymbol**	**Description**	**EntrezIDs**	**EntrezID**
Antxr2	Anthrax toxin receptor 2	305633	305633
Bmp2k	BMP2 inducible kinase	NA	NA
Btc	Betacellulin	64022	64022
Lypd1	Ly6/Plaur domain containing 1	360838	360838
Csrp2	Cysteine and glycine-rich protein 2	29317	29317
Rnd3	Rho family GTPase 3	295588	295588
Itga4	Integrin subunit alpha 4	311144	311144
Zwilch	Zwilch kinetochore protein	691493	691493
Ankh	ANKH inorganic pyrophosphate transport regulator	114506	114506
Epb41l2	Erythrocyte membrane protein band 4.1-like 2	309557	309557
Capg	Capping actin protein, gelsolin like	297339	297339
Inhba	Inhibin beta A subunit	29200	29200
Prss12	Protease, serine 12	85266	85266
Mal	Mal, T-cell differentiation protein	25263	25263
Tpm4	Tropomyosin 4	24852	24852
Hist1h1a	Histone cluster 1 H1 family member a	291145	291145
Shtn1	Shootin 1	292139	292139
Rnf40	Ring finger protein 40	266712	266712
Cuedc2	CUE domain containing 2	294009	294009
Mag	Myelin-associated glycoprotein	29409	29409
Fgf5	Fibroblast growth factor 5	60662	60662
Ctnnd1	Catenin delta 1	311163	311163
Lbh	Limb bud and heart development	683626	683626
Otud7b	OTU deubiquitinase 7B	310677	310677
Hist1h1d	Histone cluster 1, H1d	201097	201097
Hist1h1c	Histone cluster 1 H1 family member c	684681	684681
NA	NA	NA	NA
Cdkn1c	Cyclin-dependent kinase inhibitor 1C	246060	246060
LOC103692716	Heat shock protein HSP 90-alpha	299331	299331
Hist1h1b	Histone cluster 1 H1 family member b	680522	680522
Tmem176b	Transmembrane protein 176B	171411	171411
Ankrd1	Ankyrin repeat domain 1	27064	27064
Scn4b	Sodium voltage-gated channel beta subunit 4	315611	315611
Susd2	Sushi domain containing 2	294335	294335
Cbx6	Chromobox 6	315136	315136
LOC686911	Similar to exocyst complex component 1 (exocyst complex component Sec3)	686911	686911

**Table 3:** Common DE genes for both doses at 24 h. Red means upregulated DEs, whereas green shows downregulated Des.

**geneSymbol**	**Description**	**EntrezIDs**	**EntrezID**
Fzd1	Frizzled class receptor 1	58868	58868
Hacd2	3-hydroxyacyl-CoA dehydratase 2		
Cfap20	Cilia and flagella-associated protein 20	307642	307642
Rph3a	Rabphilin 3A	171039	171039
Gabra2	Gamma-aminobutyric acid type A receptor alpha2 subunit	289606	289606
Ryr2	Ryanodine receptor 2	689560	689560
Mag	Myelin-associated glycoprotein	29409	29409
Scn1a	Sodium voltage-gated channel alpha subunit 1	81574	81574

**Table 4:** FC, p, and avgRank values for other possible relevant and significant DE genes for all groups.

	**Description**	**gene**	**FC**	***P*-value**	**avgRank**
HK-1 1 μM 24 h	Myelin basic protein	Mbp	−1.64	0.037	120
	G protein-coupled receptor 108	Gpr108	1.52	2.30E−02	190
	Potassium voltage-gated channel-interacting protein 4	Kcnip4	−1.91	4.50E−02	85
HK-1 500 nM 6 h	Peripheral myelin protein 2	Pmp2	1.69	4.79E−02	318
	PACAP (pituitary adenylate cyclase-activating polypeptide)	Adcyap1	−1.74	4.5E−02	280
	Glial cell-derived neurotrophic factor	GNDF	1.84	4.42E−02	223
HK-1 500 nM 24 h	NADH:ubiquinone oxidoreductase subunit B6	Ndufb6	18.27	1.57E−06	30
	Transmembrane protein 128	Tmem128	3.59	1.26E−04	156
HK-1 1 μM 6 h	Myelin expression factor 2	Myef2	2.7	3.09E−04	105
	Fibroblast growth factor 5	Fgf5	4.91	2.03E−02	48
	Fibroblast growth factor 9	Fgf9	−3.28	1.40E−02	57
	Myelin protein zero	Mpz	2.0	3.63E−02	548
	NADH:ubiquinone oxidoreductase subunit B6	Ndufb6	−2.7	2.87E−02	304
	Glial cell-derived neurotrophic factor	GNDF	2.8	5.63E−04	101

### 3.2. Potential targets for HK-1

**Figure 2:**
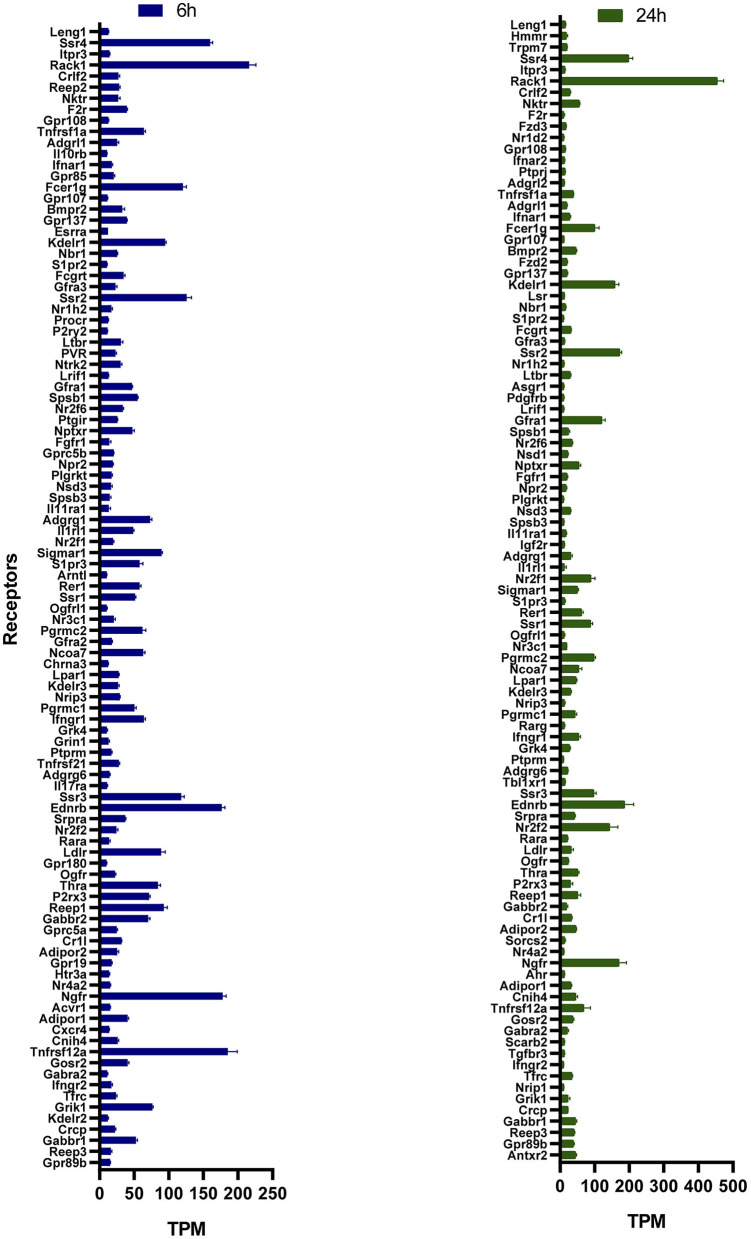
TPM values of receptors in control primary sensory neuronal cultures 6 h and 24 h. All data are shown in the Supplementary Image I1. Receptors having a TPM >10 with specific roles in neural mechanisms are demonstrated here. Functions of receptors were filtered based on public databases (https://rgd.mcw.edu/, https://www.genecards.org/, and https://www.ncbi.nlm.nih.gov).

**Figure 3:**
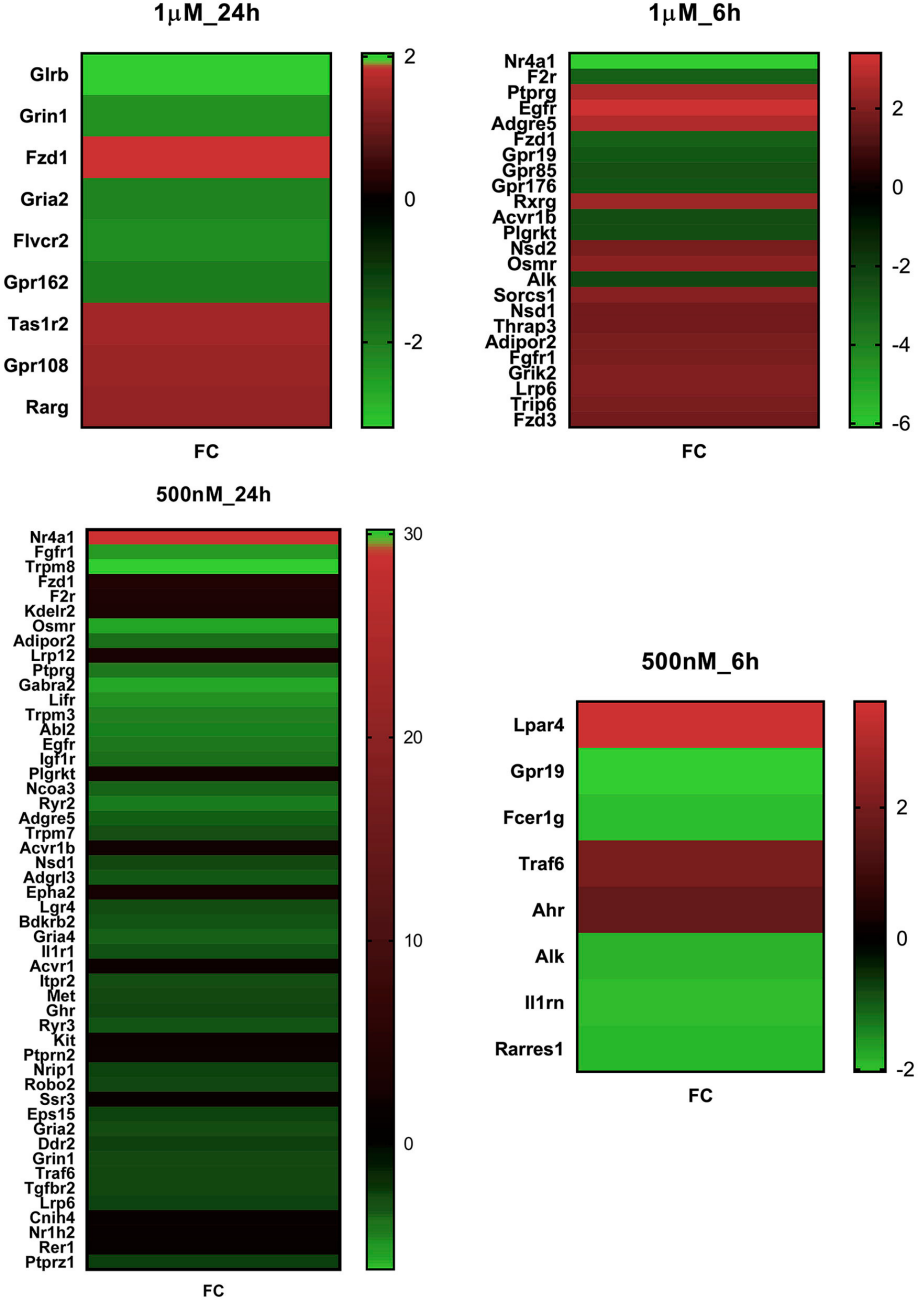
FC value of significant differentially expressed genes in primary sensory neuron cultures of the trigeminal ganglia in response to two HK-1 concentrations after 6 h and 24 h treatment durations. Abbreviations are shown in Supplementary Table S4. All DE genes can be found in Supplementary Images I2–I5.

The mRNA level of the Tacr3 receptor was detected around the detection limit, showing that low expression of Tacr1 and Tacr2 was not found in these primary cell cultures. This result was confirmed by earlier, unpublished findings with primary cell cultures from adult rat trigeminal ganglia showing similar expression patterns of these receptors. The TPM values of these receptors in different primary cultures are shown in Supplementary Table S3. No significant difference was detected between the HK-1-treated and control groups; the overlapping receptors are shown in Figure 2. To identify potential target molecules, we collected relevant receptors related to neural and/or inflammatory mechanisms based on different gene databases. Notably, the MAS-related G protein-coupled receptor, member B5 (Mrgprb5), was detected in all groups, however, with transcripts per million (TPM) below 2. Figure 3 shows receptors present in control primary sensory neuronal cultures at 6 h and 24 h. Abbreviations for receptors can be found in Supplementary Table S4. The expressions of the receptors were similar at 6 h and 24 h. Rack1, Ngfr, and Ednrb were detected at very high TPM at both sampling timepoints. Tnfrsf12a, Adipor1, and Adipor2 were also present in both concentrations. Ntrk1, P2rx3, and F2r were also expressed in both cases, highlighting the possible impact on pain transmission and calcium ion flow. Cxcr4 was found at 6 h. Figure 2 shows the DE receptors. Adipor2 at 500 nM 24 h was downregulated; F2r at 1 μM 6 h was, however, upregulated at 500 nM 24 h. Not only F2r but also Egfr expression was upregulated at 1 μM 6 h. An interesting result was the expression change in transient receptor potential melastatin 3,7,8 (Trpm3,7,8) cation channels at 500 nM 24 h. Supplementary Image I5 shows important receptors for all treatment conditions.

### 3.3. Signaling pathways influenced by HK-1 treatments

**Figure 4:**
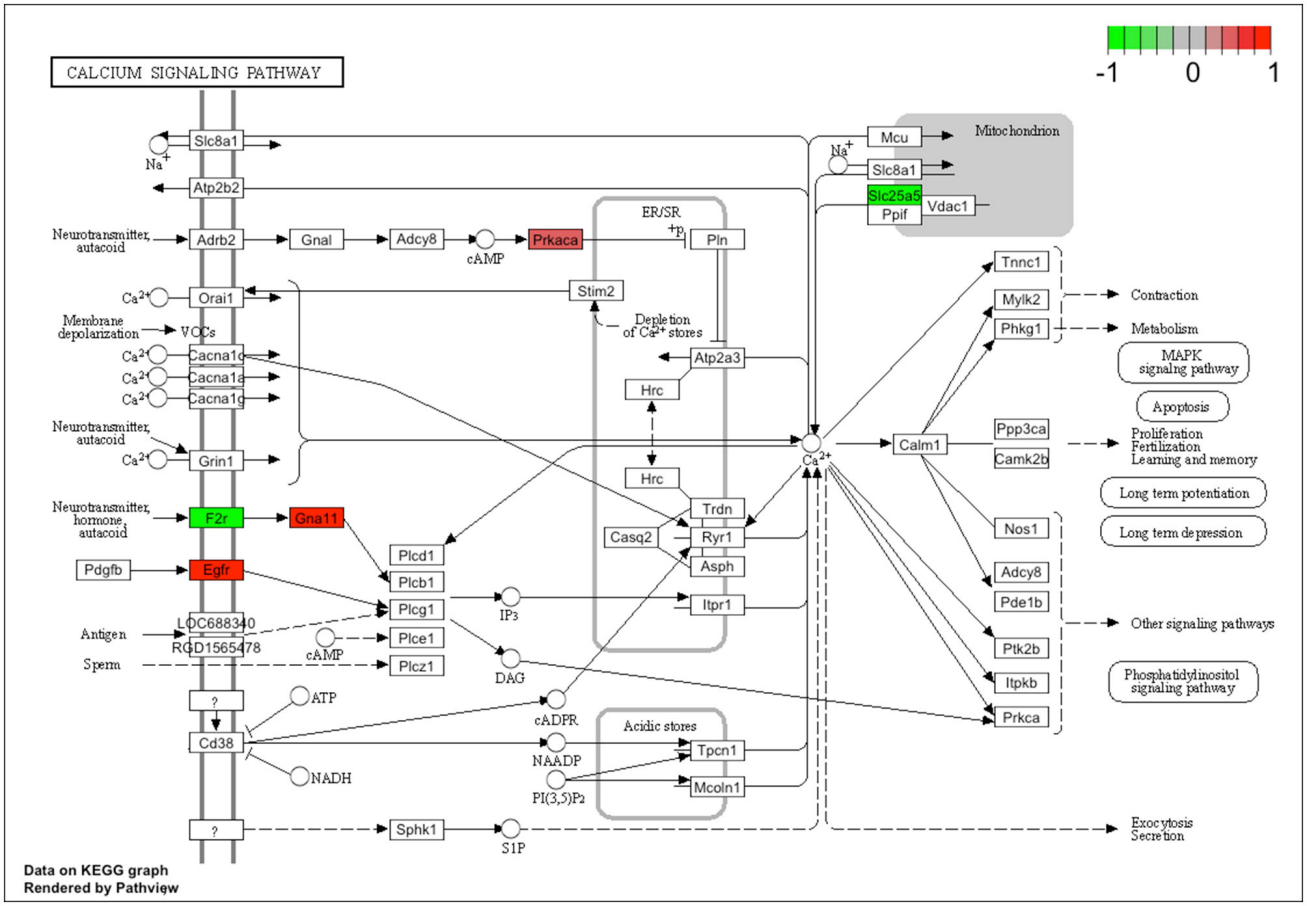
Genes involved in the calcium signaling pathway from the KEGG database. Colored rectangle is for the altered expression of genes at 6 h of HK-1 1 μM. Color means Z-score calculated from fold change value; green means downregulated, and red means upregulated.

Significantly altered pathways 6 h after 500 nM and 1 μM HK-1 treatment determined by the Kyoto Encyclopedia of Genes and Genomes (KEGG) database were predominantly related to calcium and Wnt signaling (Table 5). Figure 4 shows calcium signaling pathways with significant DE genes and represents the FC values of DE genes involved in this process. Among receptors, F2r had shown down-, Egfr upregulation. The transcriptomic level of Slc25a5 and Prkaca (protein kinase CAMP-activated catalytic subunit alpha) has changed negatively, and that of Gna11 (G protein subunit Alpha 11) and Prkacb (protein kinase cAMP-activated catalytic subunit beta) has changed positively.

**Table 5:** Pathways from the KEGG database for HK-1 1 μM 6h group.

**KEGG.ID**	**Term**	**Annotated**	**significant**	**Expected**	**P.Value**
4540	Gap junction	55	7	1.8588850	0.002
4530	Tight junction	70	7	2.3658537	0.009
4520	Adherens junction	52	6	1.7574913	0.007
4962	Vasopressin-regulated water reabsorption	33	5	1.1153310	0.004
4916	Melanogenesis	51	6	1.7236934	0.007
5218	Melanoma	40	5	1.3519164	0.010
4020	Calcium signaling pathway	67	6	2.2644599	0.024
4912	GnRH signaling pathway	57	5	1.9264808	0.042
4310	Wnt signaling pathway	91	7	3.0756098	0.032
4730	Long-term depression	38	4	1.2843206	0.038
4914	Progesterone-mediated oocyte maturation	57	5	1.9264808	0.042
5200	Pathways in cancer	188	11	6.3540070	0.049

Significant column shows the number of DE genes from annotated genes of a given pathway. Pathway was altered significantly if a p-value of ≤ 0.05. Pathways, having a Significant score ≥ 4 were thought of as interesting findings.

The Gene Ontology (GO) database provides information on the functions of genes. Tables 6A–D shows the collected GO terms for all treated groups, simplified with the Revigo tool. Table 6A shows GO results for the HK-1 1 μM 6 h group. Protein kinase inhibitor activity, protein kinase A regulatory subunit binding, and thyroid hormone receptor binding are present in this list. Synaptic cleft was found to be a significant GO term in the 6 h results independent of the HK-1 concentration. Interestingly, biological processes altered by 500 nM HK-1 at 6 h (Table 6B) included the adenylate cyclase-activating G protein-coupled receptor signaling pathway, positive regulation of T cell-mediated immunity, neutrophil chemotaxis, positive regulation of leukocyte adhesion to vascular endothelial cells, and highlighting possible immunological functions. Notable cellular components were the synaptic cleft, T-tubule, and myelin sheath, and remarkable molecular functions were adrenergic receptor binding, NADH dehydrogenase activity, and cAMP binding. GO results revealed that 1 μM HK-1 treatment significantly altered the glutamate and insulin receptor signaling pathways at the 24-h timepoint, which are intracellular responses to calcium ions, as shown in Table 6C. A notable affected cellular component was the synaptic vesicle. Among modified molecular functions, we found ligand-gated ion channel activity, postsynaptic neurotransmitter receptor activity, and sodium channel activity (Table 6C). Meanwhile, 500 nM HK-1 at the same timepoint regulated presynapse assembly, cGMP-mediated signaling, mitochondrial ATP synthesis-coupled electron transport, Schwann cell proliferation, cell fate specification, positive regulation of dendritic spine development, positive regulation of cold-induced thermogenesis, and ceramide biosynthetic processes. As significant molecular functions, palmitoyltransferase activity, fibroblast growth factor binding, oxidoreductase activity, and acting on peroxide as an acceptor were found (Table 6D).

**Table 6:** A–D. GO terms for each group merged with the Revigo tool.

**6h HK-1 1μM**	**GO ID**	**Name**	**Value (logP)**
**(A)**			
**Biological processes**	GO:0007062	Sister chromatid cohesion	−4.2
	GO:0007063	Regulation of sister chromatid cohesion	−3.7
	GO:0048538	Thymus development	−2.8
	GO:0080182	Histone H3-K4 trimethylation	−2.0
	GO:1903429	Regulation of cell maturation	−1.9
	GO:0051973	Positive regulation of telomerase activity	−2.5
	GO:0008589	Regulation of smoothened signaling pathway	−1.9
	GO:0071108	Protein K48-linked deubiquitination	−1.9
	GO:0038083	Peptidyl-tyrosine autophosphorylation	−1.9
	GO:0045992	Negative regulation of embryonic development	−1.9
	GO:0035019	Somatic stem cell population maintenance	−1.9
**Cellular component**	GO:0000786	Nucleosome	−2.6
	GO:0043083	Synaptic cleft	−1.8
	GO:0014704	Intercalated disc	−1.3
	GO:0099738	Cell cortex region	−2.0
	GO:0045120	Pronucleus	−1.7
	GO:0031519	PcG protein complex	−1.6
	GO:0000307	Cyclin-dependent protein kinase holoenzyme complex	−1.6
	GO:0044815	DNA packaging complex	−2.1
	GO:0005868	Cytoplasmic dynein Complex	−1.4
	GO:0004860	Protein kinase inhibitor activity	−1.9
	GO:0018024	Histone-lysine N-methyltransferase activity	−1.5
**Molecular function**	GO:0034237	Protein kinase A regulatory subunit binding	−2.5
	GO:0048027	mRNA 5'-UTR binding	−2.2
	GO:0042562	Hormone binding	−1.5
	GO:0090079	Translation regulator activity, nucleic acid binding	−2.0
	GO:0031490	Chromatin DNA binding	−1.6
	GO:0046966	Thyroid hormone receptor binding	−1.4
	GO:0070182	DNA polymerase binding	−1.9
	GO:0051018	Protein kinase A binding	−1.9
**6h HK-1 500 nM**	**GO ID**	**Name**	**Value (logP)**
**(B)**			
**Biological process**	GO:0016584	Nucleosome positioning	−3.7
	GO:0035627	Ceramide transport	−2.5
	GO:0060788	Ectodermal placode formation	−3.5
	GO:0007189	Adenylate cyclase-activating G protein-coupled receptor signaling pathway	−3.6
	GO:0046464	Acylglycerol catabolic process	−3.2
	GO:0002711	Positive regulation of T cell-mediated immunity	−2.4
	GO:0010801	Negative regulation of peptidyl-threonine phosphorylation	−3.0
	GO:0055093	Response to hyperoxia	−2.5
	GO:0030593	Neutrophil chemotaxis	−3.2
	**GO:0019226**	**Transmission of nerve impulse**	**−2.5**
	**GO:0055094**	**Response to lipoprotein particle**	**−2.4**
	**GO:0035094**	**Response to nicotine**	**−2.4**
	**GO:0043537**	**Negative regulation of blood vessel endothelial cell migration**	**−2.6**
	**GO:0071696**	**Ectodermal placode development**	**−3.4**
	**GO:1904996**	**Positive regulation of leukocyte adhesion to vascular endothelial cell**	**−2.3**
**Cellular component**	GO:0000786	Nucleosome	−3.9
	GO:0043083	Synaptic cleft	−3.0
	GO:0030315	T-tubule	−3.0
	GO:0043209	Myelin sheath	−3.0
	GO:0099092	Postsynaptic density, intracellular component	−3.0
	GO:0044815	DNA packaging complex	−3.2
**Molecular function**	GO:0004112	Cyclic-nucleotide phosphodiesterase activity	−2.1
	GO:0030296	Protein tyrosine kinase activator activity	−2.2
	GO:0031690	Adrenergic receptor binding	−4.4
	GO:0120013	Lipid transfer activity	−1.9
	GO:0097001	Ceramide binding	−3.5
	GO:0016835	Carbon–oxygen lyase activity	−1.5
	GO:0031490	Chromatin DNA binding	−2.5
	GO:0003954	NADH dehydrogenase activity	−1.4
	GO:0030552	cAMP binding	−2.2
	GO:0030551	Cyclic nucleotide binding	−1.9
	GO:0043394	Proteoglycan binding	−1.4
**24h HK-1 1** μ**M**	**GO ID**	**Name**	**Value (logP)**
**(C)**			
**Biological process**	GO:0007215	Glutamate receptor signaling pathway	−2.7
	GO:0007606	Sensory perception of chemical stimulus	−2.8
	GO:0046834	Lipid phosphorylation	−2.2
	GO:0046626	Regulation of insulin receptor signaling pathway	−1.8
	GO:0045639	Positive regulation of myeloid cell differentiation	−1.8
	GO:0014075	Response to amine	−2.0
	GO:0097366	Response to bronchodilator	−1.9
	GO:0071277	Cellular response to calcium ion	−1.8
	GO:0050868	Negative regulation of T cell activation	−1.8
**Cellular component**	GO:0098797	Plasma membrane protein complex	−2.3
	GO:0008021	Synaptic vesicle	−1.6
**Molecular function**	GO:0005085	Guanyl-nucleotide exchange factor activity	−1.9
	GO:0005544	Calcium-dependent phospholipid binding	−2.5
	GO:0015276	Ligand-gated ion channel activity	−4.4
	GO:0030594	Neurotransmitter receptor activity	−3.4
	GO:0098960	Postsynaptic neurotransmitter receptor activity	−3.7
	GO:0005125	Cytokine activity	−1.9
	GO:0005272	Sodium channel activity	−2.9
**HK-1 500 nM at 24 h**	**GO ID**	**Name**	**Value (logP)**
**(D)**			
**Biological process**	GO:0051973	Positive regulation of telomerase activity	−3.3
	GO:0097066	Response to thyroid hormone	−2.9
	GO:1903537	Meiotic cell cycle process involved in oocyte maturation	−2.5
	GO:0080182	Histone H3-K4 trimethylation	−2.4
	GO:0050432	Catecholamine secretion	−1.5
		chromosome condensation	−2.4
	GO:0030261	Regulation of the meiotic cell cycle process involved in oocyte maturation	−2.7
	GO:1903538	Negative regulation of stem cell differentiation	−2.2
	GO:2000737	Regulation of presynapse assembly	−2.0
	GO:1905606	Regulation of anion transmembrane transport	−1.8
	GO:1903959	cGMP-mediated signaling	−2.3
	GO:0019934	Mitochondrial ATP synthesis-coupled electron transport	−2.1
	GO:0042775	Somatic stem cell population maintenance	−1.7
	GO:0035019	Positive regulation of coagulation	−2.3
	GO:0050820	Protein homotrimerization	−1.7
	GO:0070207	Receptor recycling	−1.4
	GO:0001881	Schwann cell proliferation	−2.5
	GO:0014010	Negative regulation of the BMP signaling pathway	−1.6
	GO:0030514	Endodermal cell differentiation	−1.5
	GO:0035987	Regulation of translational initiation	−1.5
	GO:0006446	Cell fate specification	−1.5
	GO:0001708	Negative regulation of chromatin organization	−1.8
	GO:1905268	Cerebral cortex cell migration	−2.2
	GO:0021795	DNA methylation or demethylation	−2.3
	GO:0044728	Protein palmitoylation	−1.8
	GO:0018345	Positive regulation of dendritic spine development	−1.6
	GO:0060999	Presynapse assembly	−1.4
	GO:0099054	Regulation of phospholipase activity	−2.3
	GO:0010517	Positive regulation of neurotransmitter transport	−1.6
	GO:0051590	Regulation of stem cell differentiation	−1.8
	GO:2000736	Regulation of cell maturation	−1.6
	GO:1903429	Positive regulation of cold-induced thermogenesis	−1.5
	GO:0120162	Ceramide biosynthetic process	−1.5
**Cellular component**	GO:0046513	Nucleosome	−4.5
	GO:0000786	DNA packaging complex	−3.8
	GO:0044815	Cytoplasmic dynein complex	−1.9
	GO:0005868	Polysomal ribosome	−1.5
	GO:0042788	Cyclin-dependent protein kinase holoenzyme complex	−1.4
**Molecular function**	GO:0000307	Antioxidant activity	−1.6
	GO:0016209	Palmitoyltransferase activity	−2.6
	GO:0016409	Fibroblast growth factor binding	−2.2
	GO:0017134	Kinesin binding	−1.9
	GO:0019894	Translation initiation factor binding	−1.6
	GO:0031369	Cadherin binding	−1.4
	GO:0045296	3',5'-cyclic-nucleotide phosphodiesterase activity	−2.6
	GO:0004114	Oxidoreductase activity, acting on peroxide as acceptor	−1.4

### 3.4. HK-1-induced signaling pathways

Table 7 shows results gained from the pathway database Reactome with DE genes for HK-1 1 μM 6 h, 500 nM 6 h, and 24 h groups. There was no significant finding for the HK-1 1 μM 24 h group. Noteworthy terms for HK-1 1 μM 6 h were apoptosis, signal amplification, programmed cell death, G alpha (s) signaling events, chaperone-mediated autophagy, and opioid signaling. ADP signaling through P2Y purinoreceptor 12 and opioid signaling were also important results for HK-1 500 nM 6 h group. Additional relevant terms for HK-1 500 nM 6 h treatment group were oxidative stress-induced senescence and GABA receptor activation. Common results for two concentrations at 6 h are the G alpha (s) signaling events, showing potential concentration- and calcium influx-independent effects of HK-1. Moreover, GPCR, GPCR ligand binding, and glycosphingolipid metabolism are important terms for HK-1 500 nM 6 h.

**Table 7:** Reactome results for HK-1 1μM 6h (A), HK-1 500 nM 24h (B), and HK-1 500 nM 6h (C).

**Reactome ID**	**Term**	**Gene ratio**	***P-*value**
**(A)**			
R-RNO-2559584	Formation of senescence-associated heterochromatin Foci (SAHF)	6/12	1.1E-06
R-RNO-2559586	DNA damage/telomere stress-induced senescence	6/30	4.3E-04
R-RNO-9009391	Extra-nuclear estrogen signaling	6/40	0.002
R-RNO-109581	Apoptosis	7/54	0.002
R-RNO-392518	Signal amplification	4/17	0.002
R-RNO-69231	Cyclin D-associated events in G1	5/28	0.002
R-RNO-69236	G1 Phase	5/28	0.002
R-RNO-75153	Apoptotic execution phase	5/29	0.002
R-RNO-163685	Integration of energy metabolism	6/43	0.003
R-RNO-5357801	Programmed cell death	7/58	0.003
R-RNO-2500257	Resolution of sister chromatid cohesion	8/75	0.004
R-RNO-418555	G alpha (s) signaling events	5/33	0.005
R-RNO-392170	ADP signaling through P2Y purinoceptor 12	3/12	0.007
R-RNO-5223345	Miscellaneous transport and binding events	3/12	0.007
R-RNO-202040	G protein activation	3/13	0.009
R-RNO-400042	Adrenaline and noradrenaline inhibit insulin secretion	3/13	0.009
R-RNO-418592	ADP signaling through P2Y purinoceptor 1	3/13	0.009
R-RNO-428930	Thromboxane signaling through TP receptor	3/13	0.009
R-RNO-9613829	Chaperone-mediated autophagy	3/13	0.009
R-RNO-111885	Opioid signaling	5/38	0.009
**(B)**			
R-RNO-432040	Vasopressin regulates renal water homeostasis via Aquaporins	4/15	6.00E-04
R-RNO-445717	Aquaporin-mediated transport	4/15	6.00E-04
R-RNO-163685	Integration of energy metabolism	6/43	0.001
R-RNO-8939211	ESR-mediated signaling	8/76	0.001
R-RNO-9018519	Estrogen-dependent gene expression	5/33	0.002
R-RNO-111885	Opioid signaling	5/38	0.004
R-RNO-2559584	Formation of senescence-associated heterochromatin foci (SAHF)	3/12	0.004
R-RNO-392170	ADP signaling through P2Y purinoceptor 12	3/12	0.004
R-RNO-202040	G protein activation	3/13	0.005
R-RNO-400042	Adrenaline and noradrenaline inhibit insulin secretion	3/13	0.005
R-RNO-2559583	Cellular senescence	7/77	0.005
R-RNO-199977	ER to Golgi anterograde transport	8/100	0.006
R-RNO-9006931	Signaling by nuclear receptors	8/100	0.006
R-RNO-211000	Gene silencing by RNA	5/43	0.006
R-RNO-112409	RAF-independent MAPK1/3 activation	3/15	0.007
R-RNO-8936459	RUNX1 regulates genes involved in megakaryocyte differentiation and platelet function	3/15	0.007
R-RNO-2559580	Oxidative stress-induced senescence	4/29	0.008
R-RNO-75153	Apoptotic execution phase	4/29	0.008
R-RNO-2559586	DNA damage/telomere stress-induced senescence	4/30	0.009
R-RNO-6807878	COPI-mediated anterograde transport	6/67	0.010
R-RNO-392518	Signal amplification	3/17	0.011
R-RNO-2299718	Condensation of prophase chromosomes	3/18	0.013
R-RNO-977443	GABA receptor activation	3/18	0.013
R-RNO-977444	GABA B receptor activation	3/18	0.013
**(C)**			
R-RNO-2559584	Formation of senescence-associated heterochromatin Foci (SAHF)	5/12	2.35E-05
R-RNO-372790	Signaling by GPCR	16/185	3.04E-04
R-RNO-2559586	DNA damage/telomere stress-induced senescence	6/30	3.5E-04
R-RNO-388396	GPCR downstream signaling	15/181	7.48E-04
R-RNO-500792	GPCR ligand binding	7/54	0.002
R-RNO-75153	Apoptotic execution phase	5/29	0.002
R-RNO-1660662	Glycosphingolipid metabolism	4/19	0.003
R-RNO-2559583	Cellular senescence	8/77	0.003
R-RNO-190828	Gap junction trafficking	4/20	0.004
R-RNO-157858	Gap junction trafficking and regulation	4/21	0.004
R-RNO-190861	Gap junction assembly	3/12	0.006
R-RNO-418555	G alpha (s) signaling events	5/33	0.004
R-RNO-2262752	Cellular responses to stress	17/258	0.004
R-RNO-8953897	Cellular responses to external stimuli	17/260	0.005
R-RNO-5218920	VEGFR2-mediated vascular permeability	4/23	0.006

Results were relevant and significant, if FDR < 0.25 and a p-value of < 0.05. The total number of background genes was 3890. Terms are in order according to p increasing value showing less statistical significance. Date ratio shows DE genes/genes involved in the Reactome pathway.

## 4. Discussion

We demonstrate here the first transcriptomic data on the signaling pathways of HK-1 in rat primary sensory neurons, focusing on potential HK-1 targets, mechanisms of action, and DE genes involved in pain transmission and inflammation. The effect of HK-1 is complex, concentration dependent, and time dependent. Our findings support the well-described nociceptive actions of HK-1 and might explain the divergent neuronal activation processes. Since HK-1 is likely to act both on the primary sensory neurons and satellite glia cells, in this study we extract knowledge on these interactions to mimic *in vivo* conditions (Sakai et al., 2012; Theoharides et al., 2019).

Peripheral inflammatory and neuroinflammatory mechanisms resulting from complex interactions of immune cells, glial cells, and neurons play crucial roles in several diseases, including chronic pain (Siiskonen and Harvima, 2019). Itga4 and Antxr2 were upregulated for both concentrations, Tenm3 for 1 μM and Cxcl9 was downregulated after 500 nM at both timepoints. Itga4 encodes CD49d (alpha 4), one chain of very late antigen 4 (VLA-4), which belongs to adhesive molecules that activate the inflammatory process by facilitating the migration of immune cells into the central nervous system. The role of VLA-4 in genetic predisposition to chronic neuroinflammatory diseases has been demonstrated by several studies (Andreoli et al., 2007; Odoherty et al., 2007; Correia et al., 2009), supporting our findings in sensory neurons. Anthrax toxin components binding to ANTXR2, highly expressed on primary sensory neurons, were shown to inhibit inflammatory and neuropathic mechanical and thermal hyperalgesia (Yang et al., 2022). Tenm3 encodes a transmembrane protein also related to pain (Rouillard et al., 2016). Chemokines (CXCL4, CXCL9, CXCL10, and CXCL11) are important nociceptive mediators produced by neurons, microglia, and/or astroglia (Colvin et al., 2004). The CXCR3 receptor is expressed predominantly on T cells activated by CXCL9, CXCL10, and CXCL11 (Ransohoff, 2009). A CXCR3 receptor antagonist was recently shown to inhibit glia activation and neuropathic pain and enhance the effectiveness of morphine (Piotrowska et al., 2018). We showed the upregulation of Prss12, Mal, and Mag, but the downregulation of the Na_v_ beta subunit 4 (Scn4b) 6 h after both HK-1 concentrations. Motopsyn/Prss12 can activate the PAR receptor in astrocytes and trigger glutamate release to activate neuronal NMDA receptors (Lee et al., 2007; Wójtowicz et al., 2015). MAL is a protein predominantly expressed by oligodendrocytes and Schwann cells and inhibits peripheral nerve myelination (Buser et al., 2009). Voltage-gated sodium channels (Na_v_) initiate the action potential in excitable cells, and their mutations are implicated in chronic pain (Namadurai et al., 2015).

**Figure 5:**
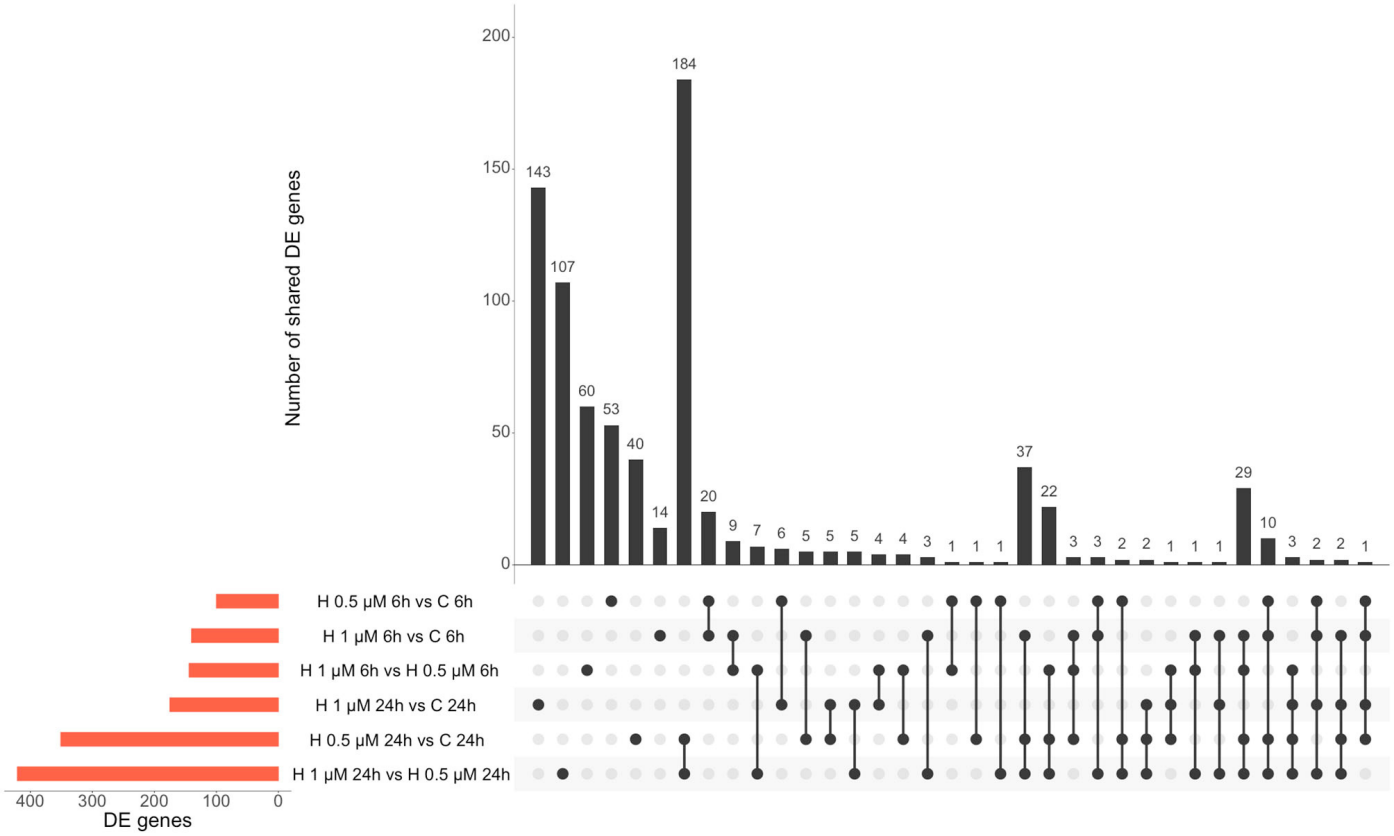
Summary of DE genes found in different treatment conditions: red columns refer to the respective group comparisons and the black columns demonstrate the shared DE genes of two or more groups. H: HK-1.

Figure 5 represents a summary of DE genes in different conditions. Both HK-1 concentrations resulted in more downregulated than upregulated genes at 24 h. Rph3a, Gabra2, Ryr2, Mag, and Scn1a were downregulated, while Hacd2 was upregulated. Hacd2 plays an important role in long-chain fatty acid biogenesis involved in neuronal membrane functions. Tachykinin receptors interact with Ryr2 in the endoplasmic reticulum, releasing calcium (Lin et al., 2005). The interaction of Rph3A with the NMDA receptor in hippocampal neurons plays a crucial role in synaptic retention and long-term potentiation. Moreover, Rph3A can also interact with AMPA receptors (Franchini et al., 2022). Notably, we have found remarkable upregulation of Fzd1 expressed by astrocytes and involved in their cross-talks (L'Episcopo et al., 2011, 2018). Increased levels of myelin-associated proteins, including Mag and Mbp proteins, were demonstrated through Activin A oligodendroglial ACVR1B-mediated white matter remyelination after ischemic stroke in mice (Zheng et al., 2021).

One of the most interesting issues is assigning potential targets for HK-1. Interestingly, the Tacr genes encoding tachykinin receptors were expressed around the detection limit in the primary sensory neuron cultures. These results are similar to the dorsal root ganglia data from Linnarson and co-workers (http://linnarssonlab.org/drg/). In human primary sensory neurons, Tac receptors were also detected at a low level (LaPaglia et al., 2018). Tachykinin receptors with three NK1R isoforms are expressed in primary sensory neurons of the rat trigeminal system (Beaujouan et al., 1999, 2002; Page, 2005; Garcia-Recio and Gascón, 2015; Edvinsson et al., 2021), which we could not detect by our sequencing technique in the cultures derived from neonatal rats. The lack of a functionally active NK1 receptor in our system is supported by no calcium influx in response to SP treatment (Borbély et al., 2020).

**Figure 6:**
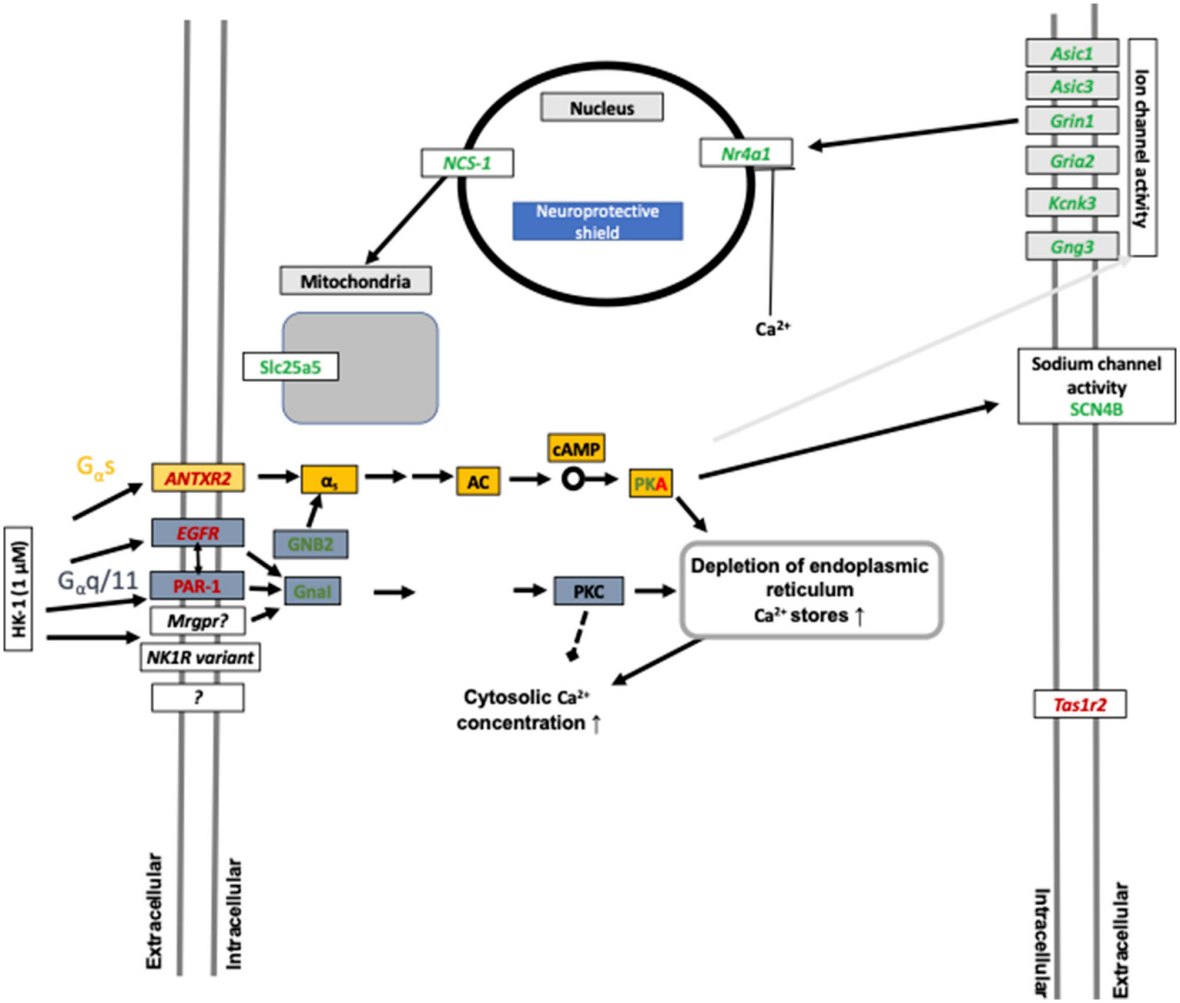
Summary of calcium influx-related gene expression changes after 1 μM HK-1 treatment. Red means upregulated, green means downregulated genes, and the gray arrow shows the 24-h effect. Protein kinase A (PKA) was up and downregulated in different concentrations. Yellow rectangular means G α s signaling, and blue means G α q/11 signaling. Gray rectangular demonstrates 24 h result. Abbreviations: G protein subunit alpha I1 (GnaI), G protein subunit beta 2 (GNB2), acid-sensing ion channel subunit 1,3 (Asic1; Asic3), potassium two pore domain channel subfamily K member 3 (Kcnk3), taste 1 receptor member (Tas1r2), adenylyl cyclase (AC), cyclic adenosine monophosphate (cAMP), G protein subunit gamma 3 (Gng3), and sodium voltage-gated channel beta subunit 4 (SCN4B) having a potential role in nociceptive sensation.

**Figure 7:**
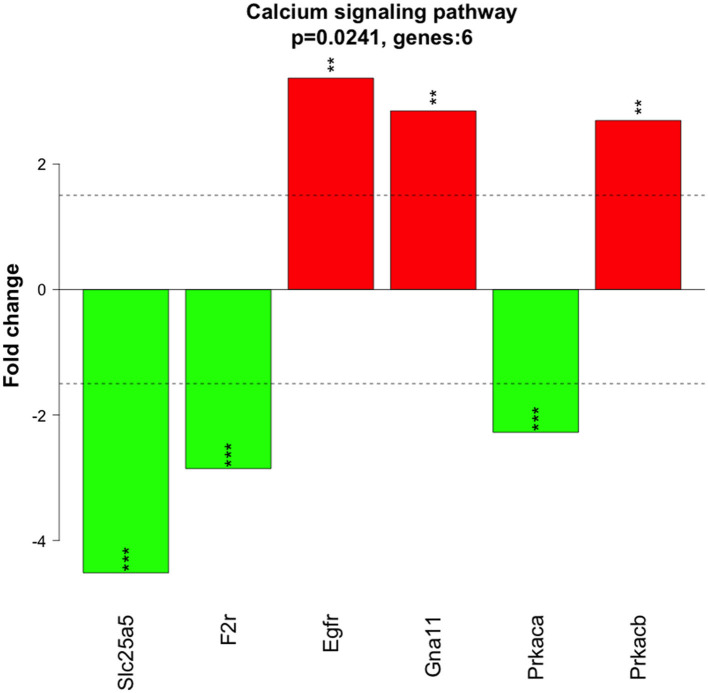
FC value of significant DE genes at 6 h of HK-1 1 μM involved in calcium signaling pathways. Red means upregulated genes and green shows downregulated genes. Abbreviations can be found in the Supplementary material. Asterisks denote these genes were found significant DE genes (*p < 0,05; **p < 0,01; ***p < 0,001) as analyzed by moderated t-test.

HK-1 at 1 μM induced calcium signaling-related transcriptomic alterations 6 h later, which is supported by our earlier fluorescent calcium influx data, which was not inhibited either by the NK1 receptor antagonist CP99994 or NK1R deletion (Borbély et al., 2020). In calcium signaling processes, F2r and EGFR receptors were DEs (Figures 6, 7). F2r/PAR1 was downregulated, while the EGFR was upregulated. NK1R signaling by SP can activate tyrosine kinase receptors such as EGFR, and PAR1 can activate EGFR (Castagliuolo et al., 2000; Arora et al., 2008). This is supported by our earlier *in vivo* findings that in HK-1-deficient mice, the PAR agonist mast cell tryptase-induced arthritic pain was decreased (Borbély et al., 2020). Mast cells were shown to be involved in fibromyalgia-related pain (Theoharides et al., 2019). Interestingly, Nr4a1 was downregulated 6 h after 1 μM and upregulated 24 h after 500 nM HK-1. Nr4a1 is an orphan nuclear receptor (Safe et al., 2016) involved in innate and adaptive immune responses in leukocytes (Shaked et al., 2015), which is downregulated and inactivated during neuroinflammation (Palumbo-Zerr et al., 2015; Shaked et al., 2015; Xiong et al., 2020). NR4a1 suppresses inflammatory responses driven by interferon (IFN) and nuclear factor kappa-light-chain-enhancer of activated B cells (NF-κB) signaling (Freire and Conneely, 2018) and microglia polarization (Rothe et al., 2017). Calcium influx might inhibit CXCL9 production and consequently decrease T cell activation (Mishra and Lal, 2021). Downregulated Slc25a5 might lead to mitochondrial dysfunction (Babenko et al., 2018), which is involved in several chronic pain conditions (Meeus et al., 2013; van den Ameele et al., 2020; Yousuf et al., 2020) related to reactive oxygen species generation and nociceptor sensitization (Salvemini et al., 2011). Intracellular calcium homeostasis, regulated mainly by the mitochondria, endoplasmic reticulum, and nucleus, determines neuronal excitability. Nuclear calcium sensor 1 (NCS-1) was suggested to modulate the expression of mitochondrial genes (Simons et al., 2019) together with the ATP sensor transporter Slc25a5 (Zhang et al., 2009). Dysfunction of Grin1, an NMDA receptor subunit, has been associated with several neurological disorders (Intson et al., 2019). Gria 2 is an AMPA receptor subunit having a major role in calcium permeation and voltage rectification; its abnormal functions also play a role in neural pathophysiologies (Salpietro et al., 2019). Downregulation of Grin1 and Gria 2 might decrease calcium concentration after a longer time, influencing neuronal survival (Guo and Ma, 2021). Several TRP channels are involved in sensory functions (Nilius et al., 2003; Voets et al., 2004, 2005; Nilius and Sage, 2005), and their expression or functional changes alter neuronal responsiveness (Nilius and Owsianik, 2011). TRPM 3,7,8 were downregulated 24 h after 500 nM HK-1, which is the opposite of what we earlier found in response to another sensory neuropeptide, PACAP (Takács-Lovász et al., 2022). Notably, PACAP was downregulated 6 h after 500 nM HK-1, suggesting a potential link between the effects of these two peptides on TRP channel expression. The mitochondrial complex I Ndufb6 subunit was significantly downregulated, similar to PACAP (Takács-Lovász et al., 2022). Figure 6 demonstrates these findings in network.

Our previous *in vivo* data demonstrated the pain-mediating role of HK-1 in several acute and chronic orofacial, neuropathic, and inflammatory pain models (partial sciatic nerve ligation, acute, and chronic CFA and mast cell tryptase-induced, as well as K/BxN serum-transfer arthritis models) (Borbély et al., 2013, 2020; Aczél et al., 2018; Hunyady et al., 2019). Although it is difficult to directly compare the present results with *in vivo* conditions, several similarities can be determined between gene expressions in the mouse TRG after adjuvant-induced orofacial inflammation and the present TRG culture experiment following HK-1 treatment. We earlier showed that the HK-1 encoding *Tac4* gene was upregulated in the TRG in the adjuvant-induced model. Meanwhile, fibroblast growth factor 13 (Fgf13, also termed as glia activating factor) was downregulated in wild-type mice, but upregulated in HK-1-deficient ones (Aczél et al., 2020). This well correlates with the Fgf9 downregulation we observed in the present study in response to 1 μM 6 h later. Fgfs are involved in neuron–glia interactions and glia proliferation, mediating neuroinflammatory mechanisms (Stork et al., 2014). Furthermore, an orphan G protein-coupled receptor, Gpr62, playing a role in axo-myelinic signaling, was upregulated in the TRG of adjuvant-treated wild-type mice (Hay et al., 2021) similar to Gpr108 upregulation after 1 μM HK-1 in the present experiment to inhibit Toll-like receptor-triggered inflammatory responses (Dong et al., 2018). The voltage-gated K^+^ channel-interacting protein 9 (Kcnj9) was also downregulated in the TRG after adjuvant injection (Aczél et al., 2020), which supports our findings on the Kcnip4 downregulations in the present model. Inward-rectifying K+ currents have been shown to change in the trigeminal ganglia during peripheral inflammation (Takeda et al., 2011). Transmembrane protein 100 (Tmem100) was upregulated in the TRG in the *in vivo* mouse model, while in our cell system the expressions of related members of this protein family, Tmem128, significantly increased. Integrin subunit alpha 7 (Itga7) involved in glia proliferation (Tan et al., 2022), was also downregulated both in the mouse model (Aczél et al., 2020) and in the present cell culture study Itgav both are expressed on glial cells (Mapps et al., 2022). RNA-Seq literature data demonstrated Kcnj9, F2rl2 (PAR3) upregulation in the DRG in a neuropathic pain model similar to what we found for F2r and PAR1 (Stevens et al., 2020). Validation of the RNA sequencing data with qPCR showed a good correlation for all investigated genes except Fgfr1. However, such discrepancies are not unusual based on the literature data; others described that these two techniques provide similar results in 87.9% (Protasio et al., 2013) and 95.2% of genes (Liu et al., 2014), which is in agreement with the present findings.

In conclusion, this is the first approach to determining transcriptomic alterations induced by HK-1 in primary sensory neurons that might be related to their activation mechanisms and pain. We showed concentration- and time-dependent actions and identified potential pain-related processes such as microglial–neuron or GPCR interactions, myelin-associated gene expression, and orphan GPCRs having roles in GABAergic pathways by modifying NMDA and AMPA receptor subunits, demonstrating that not just neurons, but mast cells and glia cells might be affected by HK-1. The effects specific to the 1 μM (sodium channel activity, neurotransmitter receptor activity, cytokine activity) were potentially calcium influx-dependent, as we earlier showed that the 1 μM, but not the 500 nM concentration, induced a calcium signal in this experimental paradigm. Although the target(s) of HK-1 cannot be determined on the basis of the present results, it is not likely to be the NK1 tachykinin receptor. Identifying the receptor might open novel perspectives in analgesic research.

## Data availability statement

The datasets presented in this study can be found in online repositories. The names of the repository/repositories and accession number(s) can be at: https://www.ebi.ac.uk/ena, PRJEB47291.

## Ethics statement

The animal studies were approved by the Ethical Committee on Use of Laboratory Animals at the University of Pécs (permission no: BA02/2000-51/2017). The studies were conducted in accordance with the local legislation and institutional requirements. Written informed consent was obtained from the owners for the participation of their animals in this study.

## Author contributions

ÉB, ÉS, KB, and ZH: conceptualization. TA and KB: methodology. JK: software. JK, KT-L, LC, and PU: validation. KT-L and JK: formal analysis, data curation, and visualization. ZH: investigation and supervision. AG and ZH: resources and funding acquisition. KT-L: writing—original draft preparation. KT-L, ÉB, ÉS, KB, and ZH: writing—review and editing. All authors contributed to the article and approved the submitted version.
