# Supplementary material for: Hemokinin-1 induces transcriptomic alterations in pain-related signaling processes in rat primary sensory neurons independent of NK1 tachykinin receptor activation
Source: Front Mol Neurosci. 2023 Oct 27;16:1186279. doi: 10.3389/fnmol.2023.1186279 (PMC10641776; doi:10.3389/fnmol.2023.1186279)
Supplement: Supplementary file 1 [file Table_1.docx]

| **Gene** | **Direction** | **Sequence (5'->3')** | **RefSeq** | **Product length (bp)** |  |
| --- | --- | --- | --- | --- | --- |
| Nr4a1 | FW | GTTGGGGTAGTGTGCGAGAAGG | NM_024388.2 | 150 |  |
| Nr4a1 | REV | CTGGGCTGGTTGCTGGTGTTC |  |  |  |
| Ndufb6 | FW | GCGTACCGCACCAGTCTCTT | NM_001106646.1 | 120 |  |
| Ndufb6 | REV | TCTGGGCTTCGTGCCAACAAT |  |  |  |
| Slc25a5 | FW | TGACACTGCAAAGGGAATGCTC | NM_057102.2 | 124 |  |
| Slc25a5 | REV | ACGGCGAACCGTGTCAAAAG |  |  |  |
| Gnb2 | FW | CTGCGCTGGACGGTTGAGT | NM_031037.2 | 109 |  |
| Gnb2 | REV | TGGGACTCTGCCAAGGGGTC |  |  |  |
| F2r | FW | GCGTTCCTATGAGACAGCCAGAATC | NM_012950.2 | 143 |  |
| F2r | REV | TTCATTTTTCTCCTCCTCATCCCCT |  |  |  |
| Fgf5 | FW | GTAGCGCGACGTTTGCTTCG | NM_001412336.1; NM_022211.2 |  |  |
| Fgf5 | REV | ACATACCACTCCCGGCCTGT |  |  |  |
| Itga4 | FW | GCTGGCTGTTCACGGGTTTG | NM_001107737.1 | 136 |  |
| Itga4 | REV | TGGAGCCATGCTAATGCCAGT |  |  |  |
| Fgfr1 | FW | CTCTGGCAGCGATACCACCT | NM_024146.2 | 145 |  |
| Fgfr1 | REV | GTATGGCGCCACAGGCCTAC |  |  |  |
|  |  |  |  |  |  |
|  |  |  |  |  |  |
|  |  |  |  |  |  |
|  |  |  |  |  |  |
| Hsp90aa1 | FW | GTGTCCCGGTGCGGTTAGTC | NM_175761.2 | 80 |  |
| Hsp90aa1 | REV | GGTTGGTCTTGGGTCTGGGT |  |  |  |
|  |  |  |  |  |  |
|  |  |  |  |  |  |
| Tubb2a | FW | GGGCAAGAGCCTTCACCTCTTC | NM_001109119.1 | 148 | (reference gene) |
| Tubb2a | REV | CCATGGTAACTGCCAGTGGGG |  |  |  |
