# Supplementary material for: Hemokinin-1 induces transcriptomic alterations in pain-related signaling processes in rat primary sensory neurons independent of NK1 tachykinin receptor activation
Source: Front Mol Neurosci. 2023 Oct 27;16:1186279. doi: 10.3389/fnmol.2023.1186279 (PMC10641776; doi:10.3389/fnmol.2023.1186279)
Supplement: Supplementary file 2 [file Table_2.docx]

| Target | Biological Set Name | Fold change average qPCR | Fold change deviation (qPCR) | Fold change Sequencing |
| --- | --- | --- | --- | --- |
| Ndufb6 | H1_6h | -1.150 | 0.038 | -2.700 |
| Itga4 | H1_6h | 3.652 | 1.042 | 7.470 |
| Gnb2 | H1_6h | -1.010 | 0.160 | -6.500 |
| F2r | H1_6h | -1.080 | 0.060 | -2.920 |
| Fgf5 | H1_6h | 2.942 | 0.568 | 4.910 |
| Hsp90aa1 | H1_6h | 1.560 | 0.206 | 4.130 |
| Gnai1 | H1_6h | 1.147 | 0.037 | 3.440 |
| Ndufb6 | H500_24h | 2.105 | 0.456 | 18.270 |
| Gnb2 | H500_24h | 1.445 | 0.482 | 6.520 |
| Nr4a1 | H500_24h | 1.783 | 0.438 | 30.210 |
| Slc25a5 | H500_24h | 2.205 | 0.672 | 15.310 |
| F2r | H500_24h | 1.726 | 0.411 | 4.010 |
| Fgfr1 | H500_24h | 1.180 | 0.258 | -4.780 |
| Fgfr1 | H1_24h | 1.385 | 0.080 | 1.024 |
| Gna1 | H1_24h | 1.365 | 0.188 | 1.11 |

N=3 samples were used in RT-qPCR for treated and control group.

Independent two-tailed t-test was used for statistical analysis, at 0,05 significant level.
