# Supplementary material for: Hemokinin-1 induces transcriptomic alterations in pain-related signaling processes in rat primary sensory neurons independent of NK1 tachykinin receptor activation
Source: Front Mol Neurosci. 2023 Oct 27;16:1186279. doi: 10.3389/fnmol.2023.1186279 (PMC10641776; doi:10.3389/fnmol.2023.1186279)
Supplement: Supplementary file 7 [file Data_Sheet_1.docx]

Supplementary Material

Hemokinin-1 induces transcriptomic alterations in pain-related signalling processes of rat primary sensory neurons independently of NK1 tachykinin receptor activation

Krisztina Takács-Lovász ^1^, Timea Aczél ^1^, Éva Borbély ^1,4 *^, Éva Szőke ^1,3,4^, Lilla Czuni^2^, Péter Urbán^2^, Attila Gyenesei^2^, Zsuzsanna Helyes^1,3,4,5^, József Kun^1,2^ and Kata Bölcskei^1^

*** Correspondence:** Éva Borbély
[eva.borbely@aok.pte.hu](mailto:eva.borbely@aok.pte.hu)

# Legend of Supplementary Figures and Tables

## Supplementary Figures

## Supplementary Figure 1. Supplementary Figure1 (Figure S1) shows receptors selected by databases according to their role in neurological diseases and TPM value. Panels represent average TPM value (n=3; SEM except for HK-1 500 nM 6 h, where n=2).

## Supplementary Figure 2. Heatmap of all DE genes at 6 h 1 µM treatment.

## Supplementary Figure 3. Heatmap of all DE genes at 6 h 500 nM treatment.

## Supplementary Figure 4. Heatmap of all DE genes at 24 h 1 µM treatment.

## Supplementary Figure 5. Heatmap of all DE genes at 24 h 500 nM treatment.

## Supplementary Tables

**Supplementary Table 1.** Used primers for genes in validation of RNA sequencing result.

**Supplementary Table 2.** Result of RT-qPCR (FC and SD) and RNA sequencing result (FC) of certain genes in different conditions.

**Supplementary Table 3.** TPM values of tachykinin receptors of treated and untreated rats (unpublished result).

**Supplementary Table 4.** Abbreviations of genes.

**Supplementary Table 5.** Normalized Data with Annotations of all conditions

**Supplementary Table 6.** Raw data of all conditions
