# Supplementary material for: Hemokinin-1 induces transcriptomic alterations in pain-related signaling processes in rat primary sensory neurons independent of NK1 tachykinin receptor activation
Source: Front Mol Neurosci. 2023 Oct 27;16:1186279. doi: 10.3389/fnmol.2023.1186279 (PMC10641776; doi:10.3389/fnmol.2023.1186279)
Supplement: Supplementary file 8 [file Image_1.pdf]

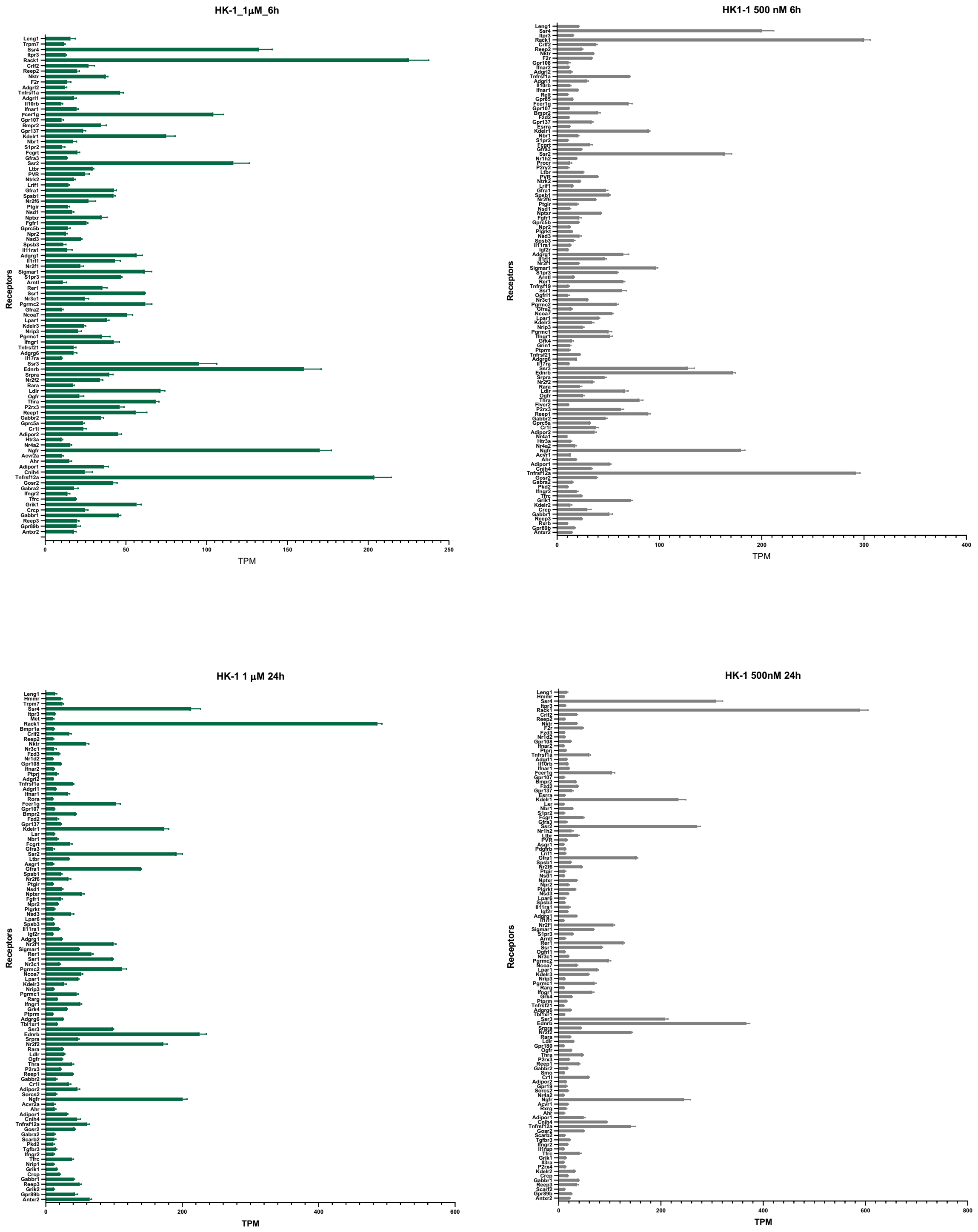

Supplementary Figure 1 (Figure S1) shows receptors selected by databases according to their role in neurological diseases and TPM value. Panels represent average TPM value (n=3; SEM except for HK-1 500 nM 6 h, where n=2).
