# Supplementary figures and images for: Hemokinin-1 induces transcriptomic alterations in pain-related signaling processes in rat primary sensory neurons independent of NK1 tachykinin receptor activation
Source: Front Mol Neurosci. 2023 Oct 27;16:1186279. doi: 10.3389/fnmol.2023.1186279 (PMC10641776; doi:10.3389/fnmol.2023.1186279)

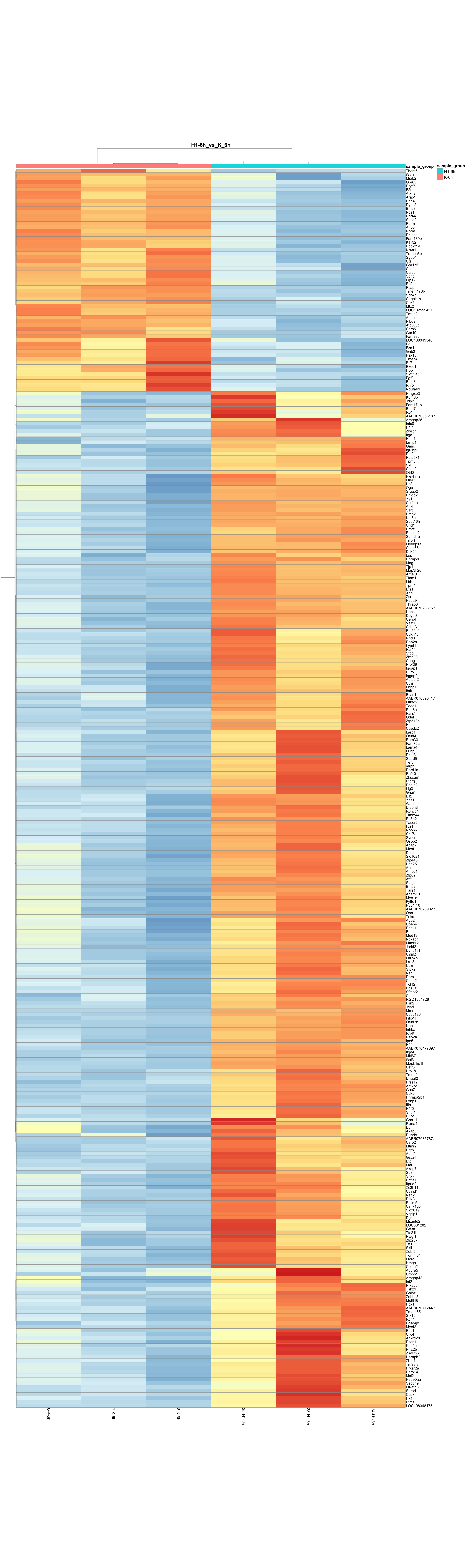

Supplement: Supplementary file 9 [file Image_2.png]

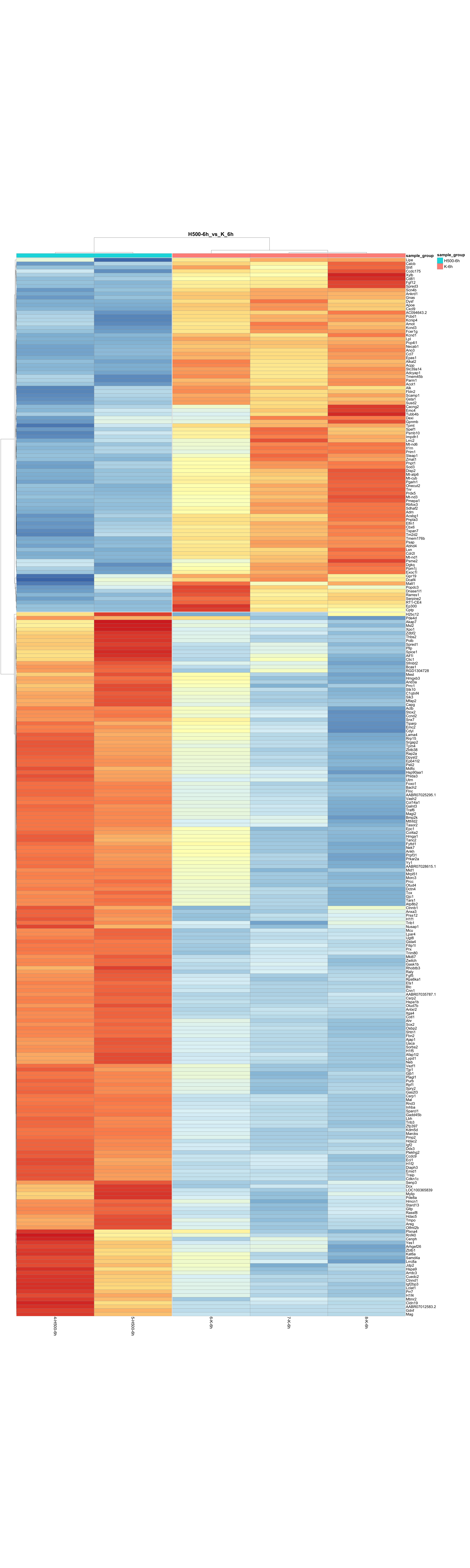

Supplement: Supplementary file 10 [file Image_3.png]

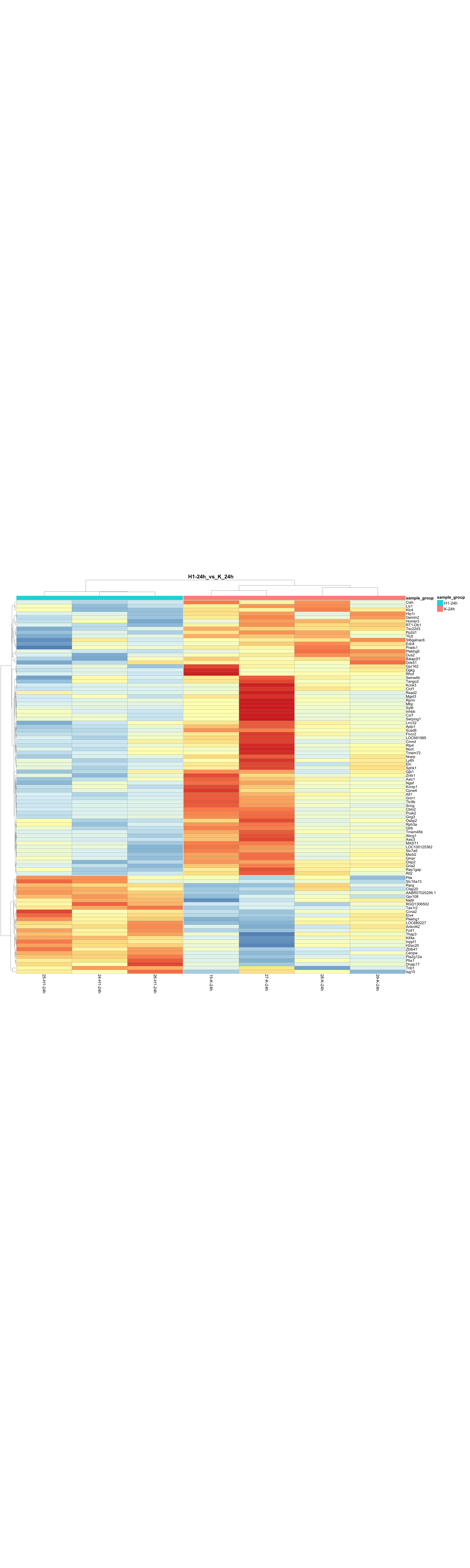

Supplement: Supplementary file 11 [file Image_4.png]

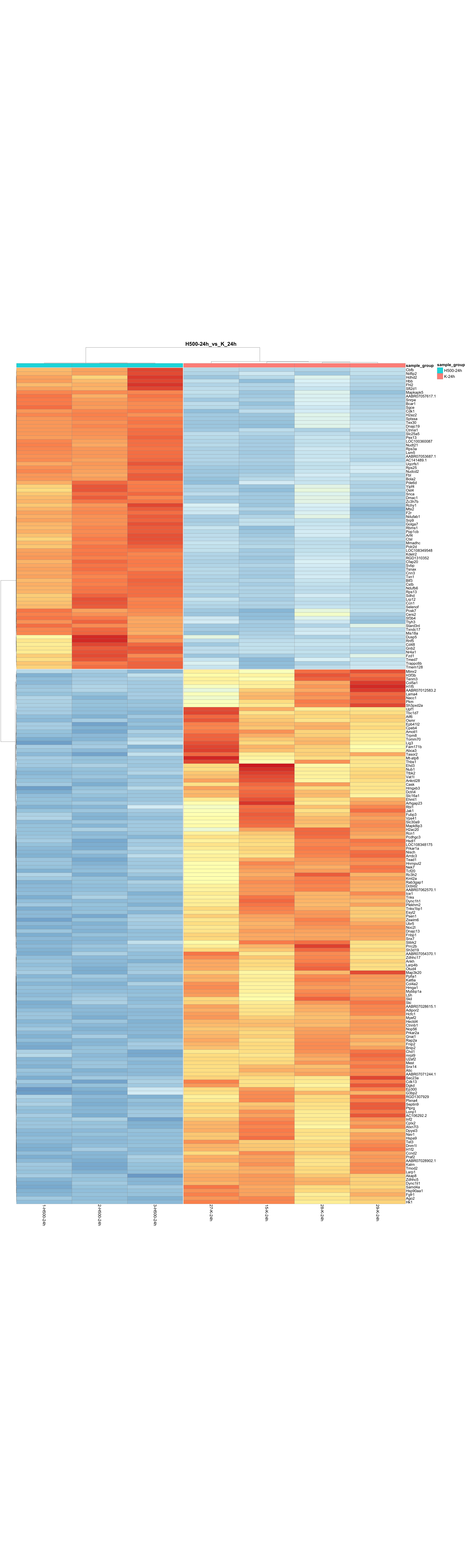

Supplement: Supplementary file 12 [file Image_5.png]
